# The slow-evolving *Acorus tatarinowii* genome sheds light on ancestral monocot evolution

**DOI:** 10.1038/s41477-022-01187-x

**Published:** 2022-07-14

**Authors:** Tao Shi, Cécile Huneau, Yue Zhang, Yan Li, Jinming Chen, Jérôme Salse, Qingfeng Wang

**Affiliations:** 1 CAS Key Laboratory of Aquatic Botany and Watershed Ecology, Wuhan Botanical Garden, Chinese Academy of Sciences, Wuhan, China; 2 Center of Conservation Biology, Core Botanical Gardens, Chinese Academy of Sciences, Wuhan, China; 3 UCA, INRAE, UMR 1095 GDEC (Genetics, Diversity & Ecophysiology of Cereals), Clermont-Ferrand, France; 4 University of Chinese Academy of Sciences, Beijing, China; 5 Sino-African Joint Research Center, Chinese Academy of Sciences, Wuhan, China

**Keywords:** Phylogenetics, Plant evolution

## Abstract

Monocots are one of the most diverse groups of flowering plants, and tracing the evolution of their ancestral genome into modern species is essential for understanding their evolutionary success. Here, we report a high-quality assembly of the *Acorus tatarinowii* genome, a species that diverged early from all the other monocots. Genome-wide comparisons with a range of representative monocots characterized *Acorus* as a slowly evolved genome with one whole-genome duplication. Our inference of the ancestral monocot karyotypes provides new insights into the chromosomal evolutionary history assigned to modern species and reveals the probable molecular functions and processes related to the early adaptation of monocots to wetland or aquatic habitats (that is, low levels of inorganic phosphate, parallel leaf venation and ephemeral primary roots). The evolution of ancestral gene order in monocots is constrained by gene structural and functional features. The newly obtained *Acorus* genome offers crucial evidence for delineating the origin and diversification of monocots, including grasses.

## Abstract (web summary)

The chromosomal-level genome assembly of a species from the earliest branching monocot, *Acorus tatarinowii*, provides evidence to trace the ancestral monocot chromosomes, and hints that the monocot ancestor might have an aquatic or wetland origin.

## Main

Monocots are one of the most diverse and dominant clades of flowering plants, accounting for approximately 21% of angiosperm species diversity^1^. This clade not only includes commonly consumed horticultural products, such as banana, garlic, asparagus and coconut, but more importantly also contains the grass/cereal family (Poaceae), which comprises almost half of monocots, with economically important species such as rice, wheat, oat, sorghum and maize. The earliest fossil record of monocots, such as *Cratolirion bognerianum*^2–4^, dates back to the Early Cretaceous, and molecular dating using fossil-calibrated phylogenetic trees suggests that the crown group of monocots can be traced back to approximately 132.4–149.1 million years ago (Ma) during the Early Cretaceous^1,5,6^. This crown group diversified almost at the same time as the magnoliids and eudicots^1,5,6^. The ancestral monocot has been proposed to have an aquatic origin because the fossil record of Alismatales has been dated back to at least the Upper Cretaceous^7,8^. In addition, fossils of some of the early-branching monocots morphologically resemble some extant members of those lineages and may, therefore, have shared similar habitats with typical submerged and amphibious aquatic species (Acorales, Alismatales and Hydatellaceae)^7,8^. However, this origin remains ambiguous because of a lack of compelling proof from either palaeontology or genetics.

Exploring genomic conservation and changes during monocot evolution in a considerable sampling of taxa can help to understand the driving factors that influenced the evolutionary trajectory of monocots in terms of gene order change during monocot diversification. Whole-genome duplications (WGDs) or polyploidizations are rampant during monocot diversification^9,10^ and have been proposed as a key mechanism driving species diversification and adaptation^11^. To what extent polyploidization and derived genome reshuffling^12,13^ may have driven monocot diversification among the flowering plants is an open question that requires sampling from early-branching lineages and in-depth surveying. Moreover, at the chromosomal level (karyotype), uncovering patterns of chromosomal fusion, fission, duplication and loss during species radiation is important for our understanding of the evolutionary processes underlying monocot species diversity. By reconstructing ancestral monocot karyotypes (AMK) and gene family history, we can further uncover some key genomic changes underlying the evolutionary success of monocots.

According to phylogenetic evidence obtained by large-scale taxonomic sampling, Acorales is sister to other orders in monocots^7,14^. Thus, similar to Amborellales for angiosperms^15^ and Ranunculales for eudicots^12,16^, Acorales species are phylogenetically critical for understanding the evolutionary history of monocots. Therefore, to better track genome evolution during the emergence of monocots, we sequenced and assembled at the chromosomal level the genome of *Acorus tatarinowii* Schott (also known as *Acorus gramineus*), a medicinal plant from wetlands and creeks in East Asia with an essential oil that has antidepressant-like effects^17,18^. Considerable comparative analysis between *Acorus* and the genomes of grasses (Poeales) and other monocot orders (such as oil palm and asparagus) allowed us to reconstruct the karyotype of the most recent common ancestor (MRCA) of all extant monocots (AMK^13,19^) and further uncover key genomic events associated with the important traits and aquatic or wetland origin of ancestral monocots.

## Results

### Genome assembly and ancient tetraploidization of *Acorus tatarinowii*

**Fig. 1:**
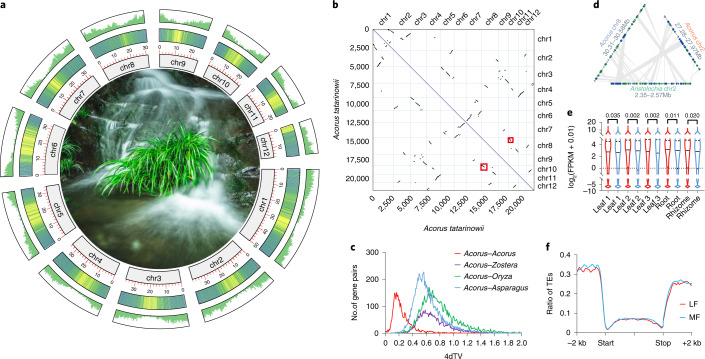
Genome assembly and a WGD of *Acorus*. **a**, Circos plot of *Acorus* genome assembly. From outer to inner circles: gene density, TE density, pseudochromosome length and *Acrorus* in an aquatic habitat. **b**, Scatter plot of *Acorus* intraspecific synteny. **c**, Density distributions of syntenic paralogues of *Acorus* and syntenic orthologues between *Acorus* and other monocots according to their 4dTV divergence. **d**, An illustration of biased subgenome fractionation between two homologous *Acorus* regions when compared with *Aristolochia*. **e**, Violin plot showing significantly higher gene expression in the LF subgenome (shown in red) than in the MF subgenome (shown in blue) for five tissues. Exact *P* values shown on the top of each violin plot are from one-sided paired *t* tests. **f**, Differences in average TE density along genes and flanking regions between duplicates residing in LF blocks and MF blocks. Source data

**Extended Data Fig. 1:**
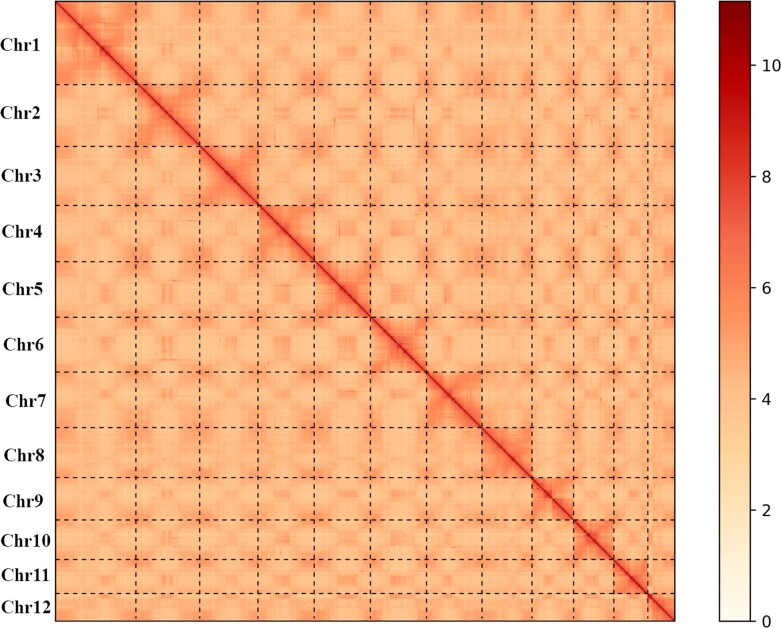
Genome-wide Hi-C interaction heatmap. Genome-wide Hi-C interaction heatmap of *Acorus* (resolution: 500 kb).

The *Acorus tatarinowii* Schott (*Acorus*) genome sequenced in this study is diploid (2*n* = 24, see http://ccdb.tau.ac.il/), with a size estimate of 470.3 Mb, an estimated heterozygosity of 0.88% and a repetitive content of 54.82%, as revealed by genomic charactor estimator analysis based on Illumina short reads (Supplementary Fig. 1). Based on PacBio, high-throughput chromosome conformation capture (Hi-C) and RNA-sequencing (RNA-seq) data, we delivered a chromosomal-level assembly and annotation of the *Acorus* genome. De novo assembly was based on 5,012,373 PacBio Sequel subreads with a total length of 110.07 Gb, a mean length of 21.96 kb and an N50 length (a metric for sequence or assembly) of 36.72 kb (Supplementary Table 1). The final 1,076 contigs covered approximately 415.18 Mb with an N50 length of 961.57 kb. Using 43 Gb of genome-wide Hi-C reads, 1,108 contigs (379.11 Mb) were anchored and ordered into 12 different pseudomolecules (Extended Data Fig. 1 and Supplementary Table 2). Among 1,614 conserved single-copy genes in BUSCO (version: embryophyta_odb10), 92.40% (1,491) of the gene set was completely retrieved, 1.4% (23) was partially retrieved and 6.2% (100) was missing. In addition, we examined the mapping rate of Illumina reads from three RNA-seq libraries and genomic DNA showing mapping percentages of 92.58%, 90.92%, 92.58% and 96.44% for young leaves, old leaves, root tissues and genomic DNA, respectively. Approximately 42.12% of the total genome assembly length was annotated as transposable elements (TEs; 174.86 Mb), of which Gypsy (13.64%), unknown long terminal repeat (10.71%) and DTM (Mutator) (DNA-type, 5.01%) accounted for the top three most abundant transposon categories (Supplementary Table 3). Combining ab initio, RNA-seq and homology-based approaches, a total of 28,241 protein-coding genes were fully annotated and densely distributed across all chromosomes, particularly where TEs were relatively scarce (Fig. 1a and Supplementary Table 1).

Based on intraspecific synteny analysis, we found large homologous blocks across all chromosomes, indicating that *Acorus* shows the remnants of one round of WGD (Fig. 1b). For example, chromosomes 8 and 10 showed strong collinearity near both chromosomal arms (Fig. 1b). Furthermore, comparison of peaks in fourfold degenerate site transversion (4dTv) distances, which represent age distributions formed by the divergence of *Acorus*–*Acorus* duplicates (4dTv median = 0.205) and the divergences of *Acorus*–*Zostera* (4dTv median = 0.666), *Acorus*–*Asparagus* (4dTv median = 0.579) and *Acorus*–*Oryza* (4dTv median = 0.741) orthologues, suggested that *Acorus* duplicates derived from a WGD after the split between *Acorus* and other monocots (two-sided Mann–Whitney *U*-test, *P* < 0.01; Fig. 1c). A comparison of synonymous substitution (*K*_S_) peaks for paralogues and orthologues confirmed that the *Acorus* WGD event is lineage-specific, making it a paleotetraploid (Supplementary Fig. 2).

**Extended Data Fig. 2:**
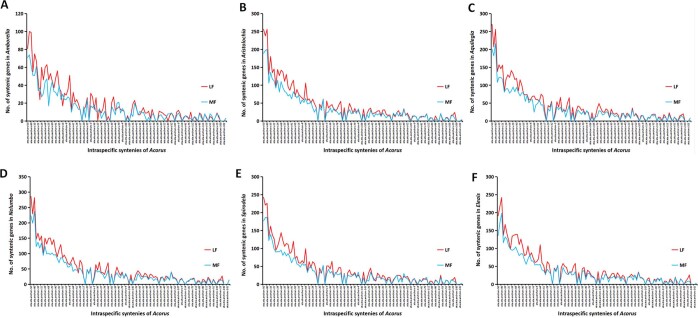
Subgenome fractionation of *Acorus* by comparing to outgroups. Differences in the number of collinear genes between LF (less fractionated) and MF (more fractionated) blocks when comparing to outgroup species, *Amborella* (A), *Aristolochia* (B), *Aquilegia* (C), *Nelumbo* (D), *Spirodela* (E) and *Elaeis* (F). Source data

To further infer the degree of subgenome fractionation and subgenome dominance in *Acorus*, we used a total of 42 monocot species genomes to classify block pairs as less-fractionated blocks (LFs) and more-fractionated blocks (MFs) based on the retention rate of ancestral genes in duplicated regions (Methods). For example, we illustrated a pair of biasedly fractionated homologous blocks of *Acorus* using *Aristolochia fimbriata* as an outgroup (Fig. 1d). Overall, most of the syntenic fragments differ in the degree to which gene duplicates are retained (retention of gene numbers), and all pairs of syntenic regions differ in length (Supplementary Table 4). To better validate and visualize LF and MF fractionation, we calculated syntenic gene retention in six independent outgroups: *Amborella trichopoda*, *Aristolochia fimbriata*, *Spirodela polyrhiza*, *Elaeis guineensis*, *Nelumbo nucifera* and *Aquilegia coerulea*. Most LFs and MFs we previously assigned had consistent fractionation bias (LF > MF in gene retention), especially for the large duplicated blocks (Extended Data Fig. 2a–f). We also found that duplicated copies of WGD genes generally showed significantly higher expression levels in LFs than in MFs for all five surveyed tissue (RNA) samples as a signature of subgenome dominance (Fig. 1e). In addition, by investigating the ratio of transposons in both genic and flanking regions, we found that TE density was significantly lower in LFs than in MFs (two-sided Mann–Whitney *U*-test, all *P* values <0.01) (Fig. 1f). Together, the biased expression and transposon density suggest subgenome dominance in *Acorus*.

### Phylogenetic positioning and genomic conservation of *Acorus*

**Fig. 2:**
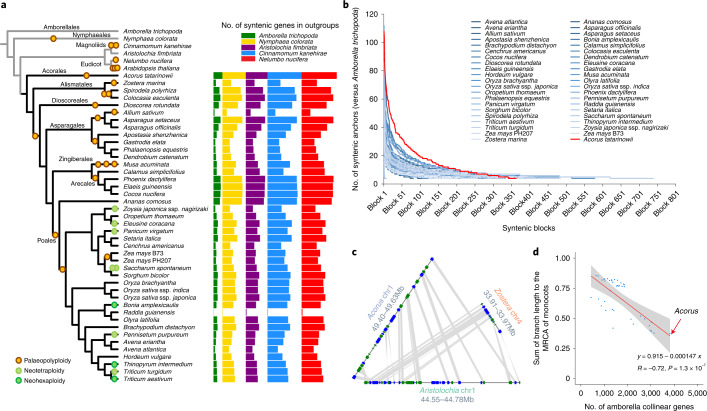
*Acorus* shows the slowest synteny loss rate and substitution rate. **a**, Distributions of syntenic gene retention from the five outgroup species in 42 monocot species shown in coloured bars. **b**, Comparison of ‘syntenic block size’ decay among different monocot species when compared with the outgroup *Amborella*. **c**, An example of stronger synteny retention in *Acorus* than in *Zestora* when compared with the outgroup *Aristolochia*. **d**, Significant negative correlation between the number of syntenic genes retained in *Amborella* and the sum of the branch lengths in the MRCA of monocots based on the concatenated single-copy gene tree for 42 monocots (blue dots). Error bands represent 95% confidence intervals based on a binomial model. Source data

**Extended Data Fig. 3:**
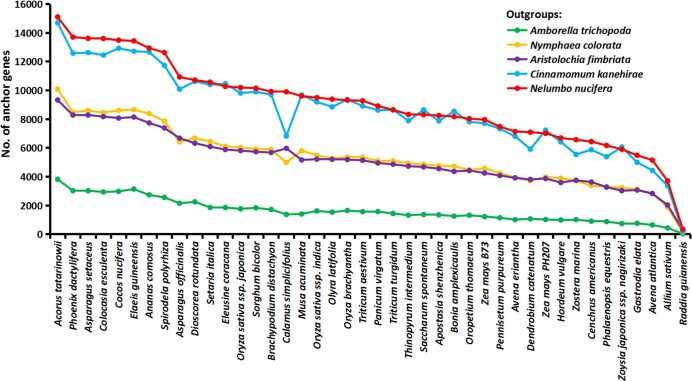
Syntenic gene retention in five outgroups. Comparison of the numbers of syntenic anchor genes in five outgroups (*Amborella*, *Nymphaea*, *Aristolochia*, *Cinnamomum* and *Nelumbo*) in relationship to monocot genome assemblies. Source data

Because *Acorus* shows genomic evidence of one single WGD, we suspect that it has a relatively conserved genome architecture within monocots. Thus, the interspecific syntenies are expected to be longer and less fragmented for *Acorus*, which it is also supposed to share more collinear genes than other monocots when compared with non-monocot genome(s). Alignment of monocot genomes to outgroup taxa with an available chromosomal-level assembly, including *Amborella trichopoda* (the earliest branching angiosperm)^15^, *Nymphaea colorata* (closely related to the Nymphaeaceae ancestral genome^20^), *Aristolochia fimbriata* (a Magnoliidae species without a WGD^21^), *Cinnamomum kanehirae* (closely related to the Magnoliidae ancestral genome^22^) and *Nelumbo nucifera* (closely related to the eudicot ancestral genome^23,24^), indicated that *Acorus* shared more collinear orthologues (anchor genes) with the outgroup genomes than all the other monocots regardless of the outgroups we used (Fig. 2a and Extended Data Fig. 3). Among these five outgroups, we found that *Nelumbo* shares the greatest number of collinear orthologues with monocots (Extended Data Fig. 3). In addition to comparison of the total number of collinear genes, we used ‘synteny decay’ to measure how rapidly the lengths of syntenic blocks decay during the divergence of two species by borrowing the philosophy of linkage disequilibrium decay^12^. Regarding the ‘decay rate’ of the syntenic block size (represented by the number of conserved anchor genes within blocks) when compared with the five outgroups, *Acorus* always showed the slowest decay rate, suggesting that its interspecific syntenies are the least fragmented within monocots (Fig. 2b and Supplementary Fig. 3). In addition, by comparing these five outgroups with *Acorus*, we found that *Nelumbo* has the slowest decay rate (Supplementary Fig. 4), in line with a report that *Nelumbo* shares the greatest number of collinear genes with monocots^24^. For example, homologous genomic regions of contrasting sizes were found between the two early-diverging monocots, *Acorus* chr1 and *Zostera* chr4, when compared with *Aristocholia* chr1 (Fig. 2c). Additionally, at a genome-wide level, the syntenic blocks between *Nelumbo* and *Acorus* are longer and more continuous than those between *Nelumbo* and rice, as we observed from the scatter plots of anchor genes along the chromosomes (Supplementary Fig. 5a,b). Thus, these pieces of evidence supported that *Acorus* has the most conserved genome architecture among all sequenced monocot genomes compared with non-monocot references (representing the Amborellales, Nymphaeales, Magnoliidae and eudicots as major clades of the early-branching flowering plants).

**Extended Data Fig. 4:**
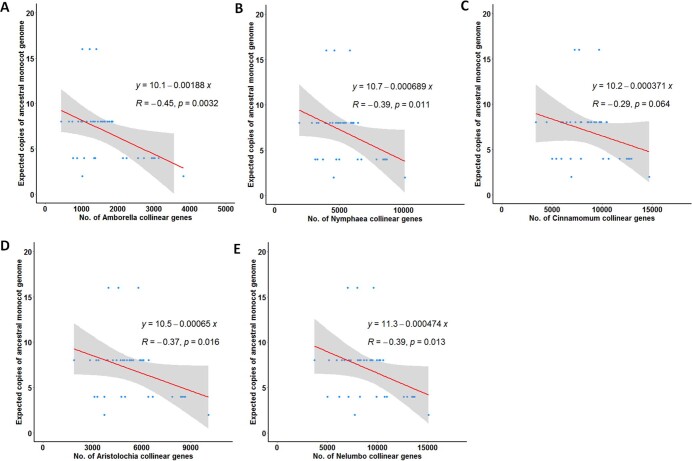
Negative correlation between syntenic gene retention and paleopolyploidies (ancient WGDs). Significantly negative correlation (calculated by Pearson’s correlation) between the number of syntenic genes in *Amborella* (A), *Nymphaea* (B), *Cinnamomum* (C), *Aristolochia* (D) and *Nelumbo* (E) and the expected copy number of genes after paleopolyploidizations. The error bands represent 95% confidence intervals based on a binomial model. Source data

Finally, we investigated what factors (such as substitution rate or ancient WGD) are associated with the synteny decay rate among monocots. Based on multiple sequence alignments of 104 single-copy orthologues, the maximum likelihood tree of monocots and outgroup taxa confirmed *Acorus* at the earliest branching position within all sequenced monocots (Fig. 2a and Supplementary Fig. 6). Notably, *Acorus* also showed the shortest sum of branch lengths from the MRCA of extant monocots, suggesting that *Acorus* is not only the earliest branching taxon (Fig. 2a), but also has the slowest sequence substitution rate among the surveyed monocot species (Supplementary Fig. 6). Furthermore, we reported that the synteny retention rates of monocots were strongly and negatively correlated with the relative sequence substitution rates and all had *P* values <0.01, indicating that rapid genome architecture change was associated with rapid sequence substitution (Fig. 2d and Supplementary Fig. 7a–d). We also showed that the synteny retention rates were negatively correlated with the number of ancient WGDs (paleopolyploidies), with *P* values of 0.0032, 0.011, 0.064, 0.016 and 0.012 for *Amborella*, *Nymphaea*, *Cinnamomum, Aristolochia* and *Nelumbo*, respectively, which were considered outgroups (Fig. 2d and Extended Data Fig. 4a–e). These results are in line with previous case studies that show extensive chromosomal rearrangements (synteny loss) after a single WGD^25–27^, as well as accelerated synteny loss with a series of WGDs. Nevertheless, we showed that there was no significant correlation between synteny loss rate and genome size, suggesting that the repetitive fraction of the genome does not significantly affect genome architecture or gene order conservation between monocots (Supplementary Fig. 8a–e).

### Biased synteny retention among different genes during monocot evolution

**Fig. 3:**
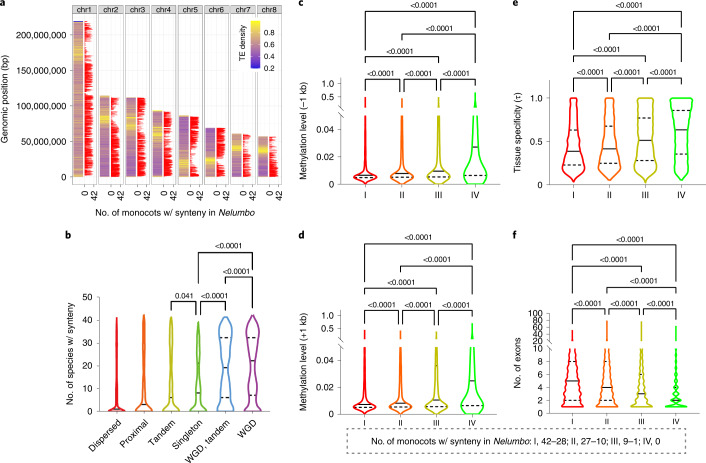
Factors associated with the distinct patterns of synteny loss in 42 monocot species based on different genes in the outgroup *Nelumbo*. **a**, The number of monocot species syntenic to *Nelumbo* (red bar) across the eight *Nelumbo* chromosomes. **b**, Violin plot of the number of monocot species syntenic to *Nelumbo* regarding gene groups of different duplication origins. **c–f**, Violin plots showing incremental changes in upstream gene methylation (**c**), downstream gene methylation (**d**), tissue specificity of expression (**e**) and exon number (**f**) for *Nelumbo* genes from group I to group IV. One-way Kruskal–Wallis test significance is shown on the top of each violin plot (adjusted *P* values). w/ synteny, with syntenic homologue. Source data

**Extended Data Fig. 5:**
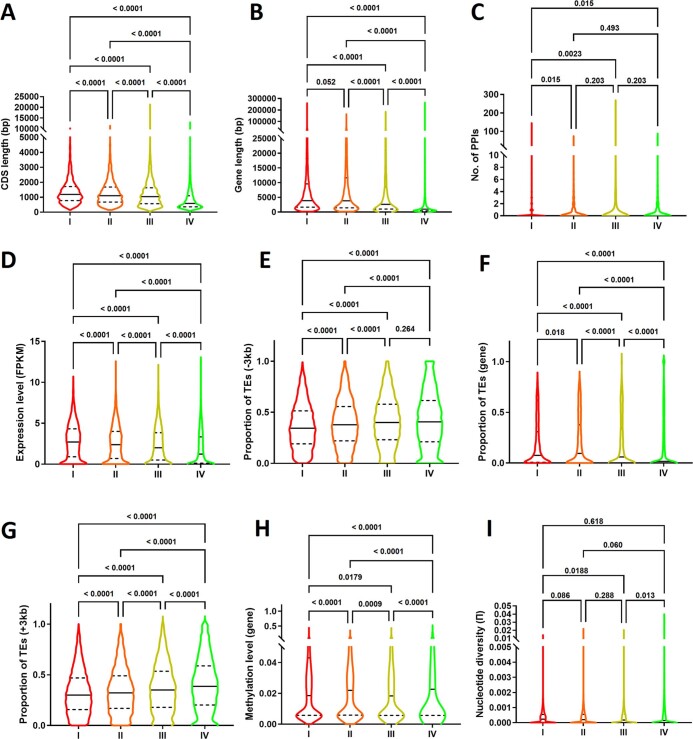
Syntenic gene retention and gene features. Violin plots showing different levels of CDS length (A), gene length (B), number of predicted protein-protein interactions (C), expression level (D), gene-upstream TE density (E), genic-region TE density (F), gene-downstream TE density (G), gene methylation (H) and nucleotide diversity (I) for *Nelumbo* genes from those with the greatest number of monocot species being syntenic (I) to those with the minimum (IV). One-way Kruskal–Wallis test significance is shown on the top of each violin plot (adjusted *P* values). Source data

To further explore the factors related to synteny retention or loss among different genes during monocot evolution, we aligned the genome of the closest outgroup (Extended Data Fig. 3 and Supplementary Fig. 4), *Nelumbo*^23^, to monocot genomes. This is because unlike other early-branching outgroups with limitations in functional and population data, *Nelumbo* offers abundant public data on gene expression from diverse organs and tissues, whole-genome methylation and population resequencing^28^. Examining this horticultural crop allowed us to gauge the variation in synteny retention rate during monocot radiation among different functional gene categories. We illustrated the rate of synteny conservation along the *Nelumbo* chromosomes and observed that the synteny retention rate was low for genes near centromeres that were enriched in TEs (Fig. 3a), putatively due to fewer genes being located near centromeres and the presence of rapid structural changes mediated by repeated sequences in these regions. We reported a difference in synteny retention depending on the status of a gene: whether it had been duplicated or not during the course of evolution^24^. We found that WGD-derived genes showed the highest retention rates, followed by ‘WGD&tandem’ genes, single-copy genes, tandem duplicates, proximal duplicates and dispersed duplicates (Fig. 3b). This result suggests that WGD, WGD&tandem genes and single-copy genes are older than those in other categories, which may reflect stronger functional constraints on these gene categories, whereas local duplicates (tandem and proximal) and dispersed duplicates are younger and under fewer structural and possibly functional constraints^24^. Despite the structural fate of syntenic genes, we also investigated their regulation, such as expression and epigenetic marks^24^. Based on the coefficient of determination *R*^2^ that measures the strength of correlation, we found that the synteny retention rate of *Nelumbo* genes in monocots is significantly correlated with gene-related traits such as the methylation level of flanking regions around genes (−1 kb and +1 kb), tissue specificity of gene expression (*τ* index), number of exons, coding sequence (CDS) length, average expression level (fragments per kilobase of exon per million reads (FPKM)), methylation level (gene), the proportion of TEs in downstream regions of genes (+3 kb), nucleotide diversity (*π*), the proportion of TEs in upstream regions of genes (−3 kb) gene length and the number of protein–protein interactions (PPIs) (Pearson correlation, *P* < 0.01); however, this rate is not correlated with the proportion of TEs in genic regions (Table 1). In parallel, to better manifest how these different factors affect the synteny retention rate, we further grouped these 29,582 *Nelumbo* genes sharing homologue(s) with at least one monocot species according to a gradual decline in the number of monocot species showing collinearity to *Nelumbo* genes and placed them into four groups (I, II, III and IV) with 6,582, 7,343, 6,561 and 9,096 genes, respectively (Fig. 3c–f). Based on pairwise comparison between the gene groups, from group I to group IV, we found incremental changes in the gene-related traits, including the methylation level of gene-flanking regions (−1 kb and +1 kb), tissue specificity of expression (*τ* index) and number of exons (Fig. 3c–f and Extended Data Fig. 5). However, such progressively changing levels from group I to group IV were not observed for all gene-related traits, including PPI, nucleotide diversity and gene methylation level (Extended Data Fig. 5), which is consistent with their relatively weaker correlations (lower *R*^2^) with synteny retention (Table 1). Collectively, these correlation tests and tendencies supported that gene-related traits, including epigenetic regulation, gene expression, gene length and exon number, which are linked to the strength of functional constraints, play crucial roles in determining gene order retention during monocot diversification.

**Table 1:** Linear regressions between the number of monocot species with a syntenic anchor to the *Nelumbo* gene (*x*) and different gene-related traits (*y*) for all *Nelumbo* genes

Gene traits (*y*)	Linear regression	*r*	*R*^2^	*P* value
Methylation level (−1 kb)	*y* = 0.0581 − 0.00111*x*	−0.22	0.0484	2.20 × 10^−16^
Methylation level (+1 kb)	*y* = 0.0517 − 0.000983*x*	−0.21	0.0441	2.20 × 10^−16^
Tissue specificity (*τ* index)	*y* = 0.571 − 0.00426*x*	−0.21	0.0441	2.20 × 10^−16^
No. of exons	*y* = 4.03 + 0.0657*x*	0.18	0.0324	2.20 × 10^−16^
CDS length	*y* = 1020 + 12.1*x*	0.17	0.0289	2.20 × 10^−16^
Average expression level (FPKM)	*y* = 2.07 + 0.0242*x*	0.15	0.0225	2.20 × 10^−16^
Methylation level (gene)	*y* = 0.0547 − 0.000709*x*	−0.15	0.0225	2.20 × 10^−16^
Proportion of TEs (+3 kb)	*y* = 0.392 − 0.00208*x*	−0.12	0.0144	2.20 × 10^−16^
Nucleotide diversity (*π*)	*y* = 0.000677 − (1.01 × 10^−5^)*x*	−0.11	0.0121	2.20 × 10^−16^
Proportion of TEs (−3 kb)	*y* = 0.423 − 0.0017*x*	−0.094	0.008836	2.20 × 10^−16^
Gene length	*y* = 7460 + 83.6*x*	0.069	0.004761	2.20 × 10^−16^
PPI	*y* = 0.871 + 0.0108*x*	0.034	0.001156	1.40 × 10^−8^
Proportion of TEs (gene)	*y* = 0.187 − 0.000149*x*	0.0081	0.00006561	0.16

*r*, correlation coefficient; *R*^2^, coefficient of determination; −1 kb or −3 kb, upstream gene regions; +1 kb or +3 kb, downstream gene regions.

### Monocot palaeohistory from the AMK

**Fig. 4:**
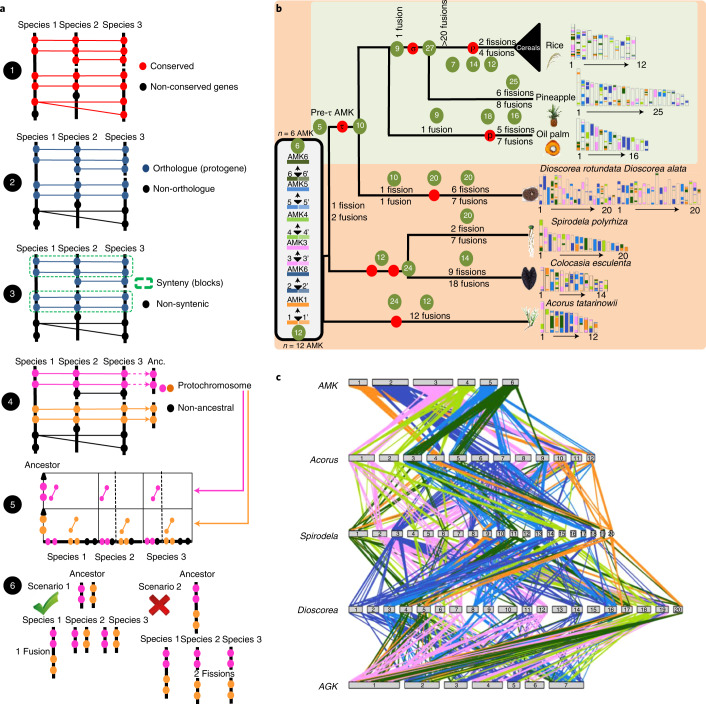
Monocot genome evolution from the inferred AMK. **a**, Illustration of the procedure for reconstructing ancestral karyotypes from conserved genes (step 1), orthologous relationships (step 2), SBs (step 3), CARs (step 4), dotplot validation (step 5) and the best scenario explaining the transition between ancestral and modern genomes (step 6). **b**, Illustration of the reconstructed AMKs (left), with a six-colour code (with light and dark shades), that evolved into the modern genomes (right) of *Acorus, Spirodela polyrhiza*, *Colocasia esculenta* and *Dioscorea* (*alata* and *rotundata*) as well as the AGK (pre-ρ and post-ρ grass ancestors), oil palm (pre-p and post-p ancestors) and pineapple as defined in Murat et al.^13^ (green panel). Polyploidization events are shown as red dots on the tree branches. The evolution of the number of chromosomes from the AMK to the extant species is shown in green circles together with inferred chromosomal rearrangements (fissions and fusions) on the tree branches. **c**, Illustration of the synteny between the AMK and modern monocot species with conserved genes linked with lines between chromosomes using the colour code from **b**.

**Extended Data Fig. 6:**
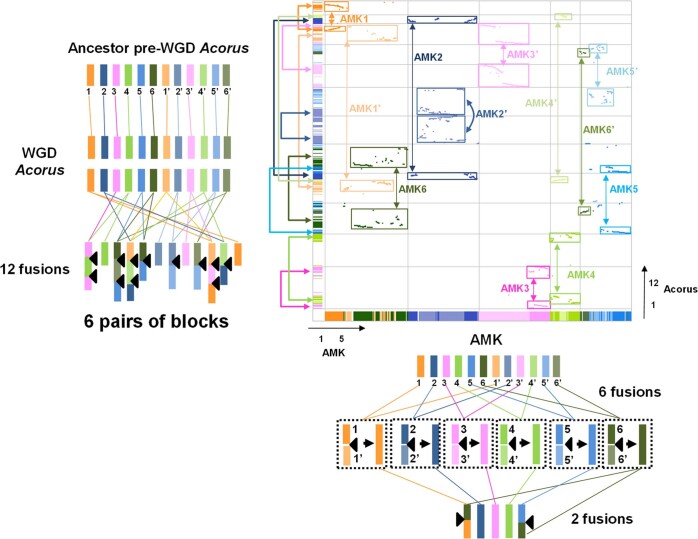
Synteny between Acorus and the *n* = 5 pre-τ AMK (from Murat et al.^13^). CENTRE-The dotplot-based deconvolution of the synteny between Acorus (*y*-axis) and the *n* = 5 pre-τ AMK (*x*-axis) defines 12 independent pairs of duplicated blocks covering the entire Acous genome (highlighted in rectangles), suggesting 12 CARs (referenced to as AMK1-1’-2-2’-3-3’-4-4’-5-5’-6-6’) at the basis of the speciation between Acorus and *n* = 5 AMK (or any species within the τ-WGD lineage). LEFT-From this ancestral state of 12 protochromosomes, the Acorus genome has been shaped through a lineage-specific WGD to reach a *n* = 24 chromosomes intermediate, followed by 12 fusions to reach the 12 modern chromosomes. BOTTOM-From this ancestral state of the 12 chromosomes, the reported *n* = 5 pre-τ AMK (Murat et al.^13^) has been shaped through 6 ancestral chromosome fusions to reach an *n* = 6 AMK intermediate (represented by six colors including orange, dark blue, pink, light green, light blue, and dark green) followed by one fission (dark green) and two fusions (dark green-orange, dark green-light blue) explaining the transition between the *n* = 6 AMK and the previously reported *n* = 5 pre-τ AMK (Murat et al.^13^) at the most recent common ancestor of Ananas, palm and grasses.

**Extended Data Fig. 7:**
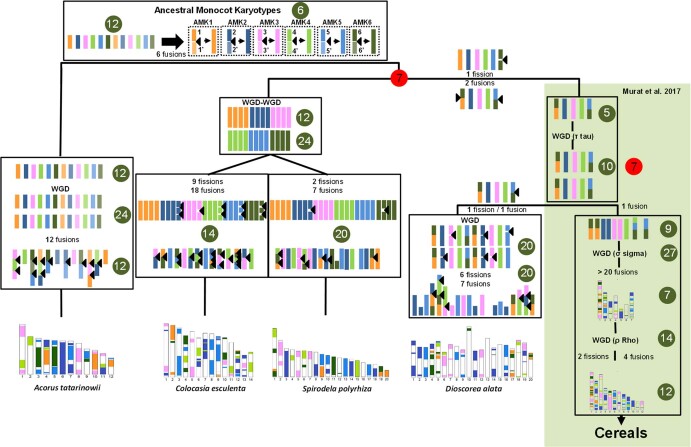
Evolutionary scenario of the monocot karyotypes. The figure illustrates the ancestral monocot karyotypes with all the proposed rearrangements (fusions, fissions) that shaped the modern genomes with the evolution of the number of chromosomes (in green circles) compared to what proposed in Xu et al.^36^ (in red circles).

**Extended Data Fig. 8:**
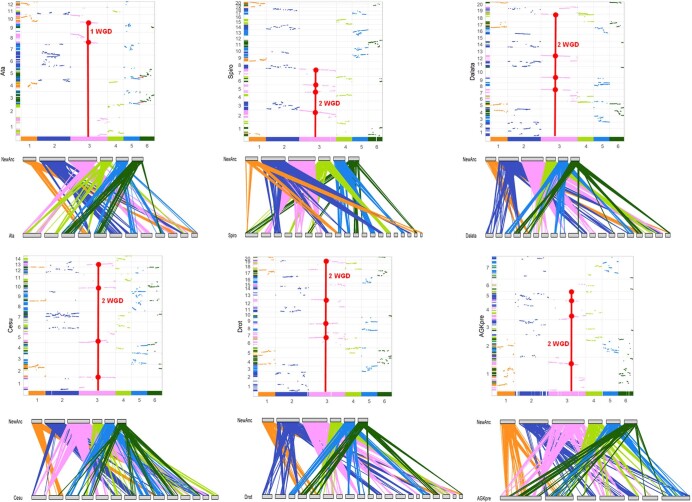
The pattern of WGDs in monocots. Dotplots illustration of the synteny between the reconstructed ancestral monocot karyotype (*n* = 6 AMK, *x*-axis) and modern species: Ata (*Acorus tatarinowii*), Cesu (*Colocasia esculenta*), Spo (*Spirodela polyrhiza*), Drot (*Dioscorea rotundata*), Dalata (*Dioscorea alata*) and AGKpre: pre-WGD (ρ) ancestral grass karyotype (*n* = 7) (*y*-axis). Signatures of reported WGD events are illuminated with red dots on the dotplots.

Access to the *Acorus* genome allowed us to investigate the AMK. From an ancestral genome that evolved into different species through speciation and distinct chromosome-shuffling events (fusion, fissions, inversions and translations), each of the ancestral chromosomes will derive a subset of extant chromosomal regions sharing synteny. Following this evolutionary evidence when reconstructing ancestral karyotypes in silico, comparative genomics of modern genomes should produce genomic fragments showing independent (non-shared) syntenic blocks, referred to as conserved ancestral regions (CARs), which are considered ancestral chromosomes in the inferred ancestral karyotype. We have proposed a six-step method for inferring ancestral karyotypes based on the comparison of extant genomes^29^ (Methods; Fig. 4a) that allowed us to previously report an AMK (hereafter referred to as the pre-τ AMK) with 5 protochromosomes and 6,707 protogenes as the MRCA of *Ananas* (pineapple)^19^, *Elaeis* (palm)^30^ and grasses (with rice, *Brachypodium* and maize as representatives of the Poaceae)^31^ (see Murat et al.^13^). This *n* = 5 pre-τ AMK evolved through a WGD event (τ) into 10 protochromosomes with 13,916 protogenes. From this *n* = 10 ancestor, the oil palm genome experienced a lineage-specific WGD event (p) and additional fusions (seven) and fissions (five) to reach the modern karyotype of 16 chromosomes. Independently, the *n* = 10 ancestor (post-τ) experienced an ancestral chromosome fusion to reach an *n* = 8 genome structure followed by a whole-genome triplication event (σ) to reach an *n* = 27 intermediate, from which pineapple (25 chromosomes) is directly inherited (with six fissions and eight fusions). This *n* = 27 ancestor (post-σ) evolved through numerous chromosomal shuffling events into the ancestral grass karyotype (AGK) with 7 chromosomes and then 12 chromosomes, following a WGD event (ρ), leading to modern grasses. The access to the current *Acorus* genome sequence and other early-branching monocot genomes, including *Spirodela polyrhiza*^32^, *Colocasia esculenta*^33^ and *Dioscorea* (*alata* and *rotundata*)^34,35^, allowed us to refine the proposed AMK genome structures earlier at the MRCA of extant monocots. In the current study, through a genome alignment (BlastP) and dotplot-based strategy (Methods) in directly extracting the catalogue of conserved genes (method step 1), one-to-one orthologous relationships (method step 2) and chromosome-to-chromosome syntenic blocks (method step 3), we performed the comparison of the genomes of *Acorus*, *Spirodela*, *Colocasia* and *Dioscorea* together with the reported *n* = 5 pre-τ AMK from Murat et al.^13^. A total of 14,404 orthologous genes (conserved between pairs of species) identified 181 syntenic blocks between *Acorus*, *Spirodela*, *Colocasia, Dioscorea* and the *n* = 5 pre-τ AMK with 2,308 single-copy protogenes, that is, genes conserved in all the investigated species (Supplementary Tables 5 and 6). To propose an updated AMK structure, we first investigated the synteny between *Acorus* and the *n* = 5 pre-τ AMK. The dotplot-based deconvolution of the synteny between the two species (method step 4) clearly defines 12 independent pairs of duplicated blocks covering the entire *Acorus* genome, suggesting 12 CARs between *Acorus* and *n* = 5 AMK (or any species within the τ-WGD lineage) (Extended Data Fig. 6). From this ancestral state, the *Acorus* genome has been shaped through a lineage-specific WGD to reach an *n* = 24 chromosome intermediate, followed by 12 fusions to reach the 12 modern chromosomes (Extended Data Fig. 6). Such dotplot-based deconvolution of the synteny between *Acorus* and the *n* = 5 pre-τ AMK clearly defines the transition between the 12 CARs that were previously defined and the *n* = 5 pre-τ AMK, introducing six ancestral chromosome fusions to reach an *n* = 6 AMK intermediate (represented by six colours, namely orange, dark blue, pink, light green, light blue and dark green in Fig. 4b) followed by one fission (dark green) and two fusions (dark green–orange, dark green–light blue in Fig. 4b) explaining the transition between the *n* = 6 AMK and the previously reported *n* = 5 pre-τ AMK at the MRCA of *Ananas*, palm and grasses (Extended Data Fig. 6). From the *n* = 6 AMK, *Colocasia* and *Spirodela* experienced two duplications to reach an *n* = 24 intermediate followed by 14 and 20 chromosomes fusions to reach their modern genome structure of 14 and 20 chromosomes, respectively. *Dioscorea* (with 20 chromosomes) is inherited directly from the *n* = 5 pre-τ AMK with seven fissions and eight fusions (Extended Data Fig. 7). The dotplot-based deconvolution of the synteny between the *n* = 6 AMK and the extant genomes validates the number of rounds of WGDs (method step 5) with one event reported in *Acorus* (Fig. 1b), and two events reported in *Spirodela*, *Colocasia* and *Dioscorea* (Fig. 4c and Extended Data Fig. 8).

Recently, Xu et al. suggested an *n* = 7 AMK before and after the ancestral τ-WGD event from the comparison of *Acorus* (*A.* *americanus*), *Spirodela*, *Colocasia, Ananas* (pineapple) and *Elaeis* (palm)^36^. We then compared our proposed *n* = 6 AMK structure with that of the seven chromosomes from Xu et al. (Supplementary Fig. 9). The two proposed AMK ancestors show a perfect chromosome-to-chromosome relationship for chromosomes 1-5, 3-4 and 5-6 between, respectively, the current *n* = 6 AMK and the *n* = 7 AMK from Xu et al.^36^. Differences are observed between the proposed AMK ancestors for chromosomes 2-(2-6), 4-(3-4) and 6-(5-7) between, respectively, the current *n* = 6 AMK and the *n* = 7 AMK from Xu et al., corresponding to different alternative scenarios proposed to explain the transition between the proposed AMKs and the modern genomes (Extended Data Fig. 7). From the proposed *n* = 6 AMK in the current study, an evolutionary scenario (method step 6) can then be inferred by taking into account the fewest number of genomic rearrangements (including inversions, deletions, fusions, fissions and translocations) that may have occurred between the AMK and modern monocot genomes (Extended Data Fig. 7). Figure 4b summarizes the number of rearrangements as well as the intermediate number of chromosomes from the AMK to the modern species investigated; in particular, when comparing *Acorus* with AMK, 12 CARs following a lineage-specific duplication occurred to create the 12 modern chromosomes. Overall, all the early-branching monocots showed far fewer mosaic fragments originating from the AMK than from the AGK, which is probably due to extensive chromosomal rearrangement (synteny loss) after multiple grass WGDs (τ, σ and ρ). Finally, our comparative genomics-based evolutionary scenario reveals the monocot palaeohistory from the AMK, with *Acorus*, sister to other extant monocots, having a karyotype most strongly resembling the AMK. Our analysis also delivers a complete catalogue of orthologues (Supplementary Table 6) between monocot genomes, which can now be used as a guide to perform translational research between the investigated species to accelerate the dissection of conserved agronomic traits.

### Biological functions at the emergence of monocots

Monocots, as a monophyletic group, process distinctive phenotypes such as parallel leaf venation, ephemeral primary roots and scattered vascular bundles in the stem; these phenotypes are similar to those of Nymphaeales but quite different from those of Amborellales, Austrobaileyales, magnoliids and eudicots^37^. To infer the functions of genes driving early monocot evolution, we built a chronogram based on 28 representative angiosperm species with fossil constraints, which predicted that the MRCA of monocots dates back to approximately 169.76 Ma, consistent with the TimeTree database (92.5–188.0 Ma)^38^ (Supplementary Fig. 10a and Supplementary Table 7). Applying the Dollo-Parsimony approach, we found that 77 and 964 orthologous groups (OGs) were acquired and lost in the AMK, respectively (Supplementary Fig. 10a and Supplementary Table 7). The 77 OGs acquired in the ancestral monocot were enriched in Gene Ontology (GO) terms such as transporter activity, plasma membrane vacuole, membrane, cell communication, transport and response to external stimulus (Supplementary Fig. 11 and Supplementary Table 8), whereas the 964 OGs lost in the ancestral monocots were enriched in GO terms such as intracellular, Golgi apparatus, mitochondrion and cytoplasm (Supplementary Fig. 12 and Supplementary Table 9). For example, OG0010560 which contains *WOX1* involved in cotyledonary primordia development, was completely lost in monocots (Supplementary Table 9). In addition, by setting a *P* value threshold of 0.05 for gene family expansion and contraction in CAFE software analysis, we extracted 41 OGs with significant expansion and 1,278 OGs with significant contraction in monocots (Supplementary Fig. 10b and Supplementary Table 7). The 41 OGs that were expanded in the ancestral monocot were enriched in GO terms such as metabolic process, cellular process and response to stress (Supplementary Fig. 13), whereas the 1,278 OGs contracted in the ancestral monocot were enriched in GO terms such as signal transduction and cell communication (Supplementary Fig. 14). For example, OG0000057, a disease resistance protein (TIR-NBS-LRR class) family, was contracted in the ancestral monocot (Supplementary Table 10), whereas OG0000047, which belongs to leucine-rich repeat protein kinases containing bacterium defence-related members, including *IOS1* and *FRK1*, was significantly expanded in the ancestral monocot (Supplementary Table 11). However, by comparing the frequency distributions of (significantly) rapidly evolving OGs detected through CAFE analysis with the OG member size (average gene copy number per species), we observed that CAFE may be insensitive to detecting significant evolutionary changes for small gene families or OGs (Supplementary Fig. 15).

**Fig. 5:**
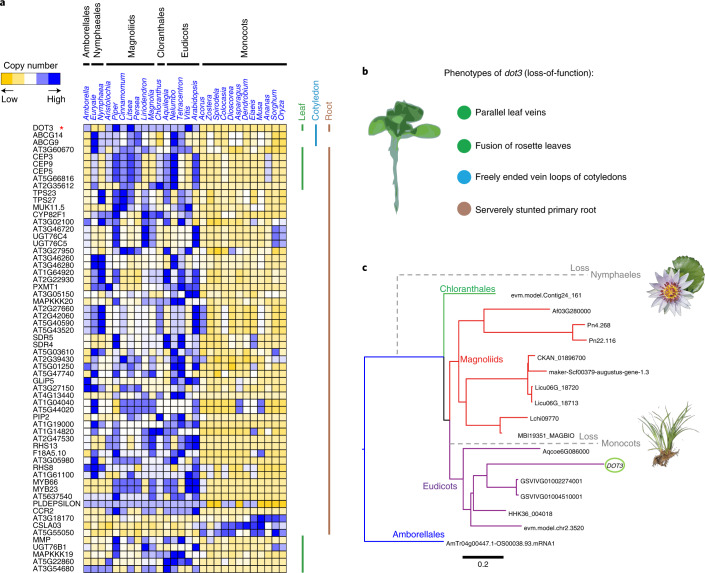
Losses of the *DOT3* gene in ancestral monocots and waterlilies associated with parallel/palmate leaf venation and ephemeral primary roots. **a**, Leaf-, root- and cotyledon-related *Arabidopsis* genes in the OGs with significant copy number differences between monocots and non-monocot angiosperms. **b**, Phenotypes of *dot3* loss-of-function mutants in *Arabidopsis* according to The Arabidopsis Information Resource records. **c**, Phylogeny of OG0011748 showing loss of *DOT3* in monocots and waterlilies. Source data

To circumvent this limit in detecting rapidly evolving OGs of smaller gene family sizes between monocots and other lineages of angiosperms, we further assigned changes based on a significant copy number difference with a *P* value threshold of <0.01 (two-sided Mann–Whitney *U-*test) and a fold change of ≥2 in the average copy number between monocots and non-monocot angiosperms (Supplementary Table 7). Among the 429 OGs with significant copy number differences between monocots and non-monocot angiosperms, 247 OGs included 607 *Arabidopsis* genes, which could be used for a deep inference of functional categories according to The Arabidopsis Information Resource annotations (Supplementary Table 12). Intriguingly, by investigating these copy number-shifting OGs based on *Arabidopsis* GO annotations related to roots, cotyledons and leaves, we found that OG0011748, containing *Arabidopsis DOT3* (*DEFECTIVELY ORGANIZED TRIBUTARIES 3*), involved in vascular tissue and primary root development^39^, showed a significant reduction in gene copy number in monocots (Fig. 5a–c). Through a detailed phylogenetic analysis of OG0011748, we found that *DOT3* was completely lost in waterlilies (*Nymphaea* and *Euryale*) and monocots, which both coincidentally showed ephemeral primary roots and palmate/parallel venation^37^ (Fig. 5c). The single-copy gene *dot3* (loss-of-function) mutants exhibited severely stunted primary roots, fusion of rosette leaves, freely ending vein loops in the cotyledons and parallel veins in *Arabidopsis* (Fig. 5b)^39^, which seem to be similar to phenotypes observed in monocots and waterlilies (Nymphaeales); therefore, their losses probably contribute to the unique leaf venation and root phenotypes in these two groups.

**Fig. 6:**
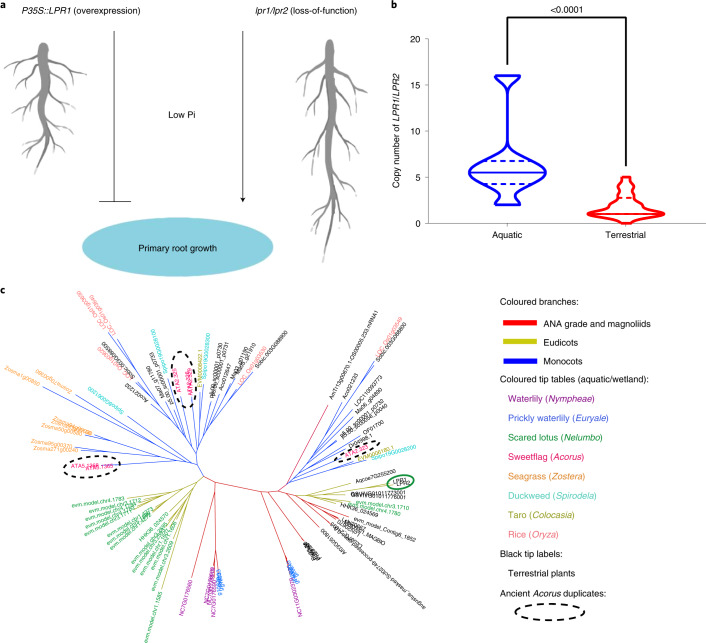
Duplications of the *LPR1*/*LPR2* family in the ancestral monocot associated with adaptation to an aquatic lifestyle. **a**, Illustration of the functional role of *LPR1*/*LPR2* in *Arabidopsis* root growth under low Pi conditions according to previous studies. **b**, There is a significantly higher copy number of *LPR1/LPR2* in aquatic plants than in terrestrial plants. Two-sided Mann–Whitney *U*-test significance is shown on the top of each violin plot (exact *P* value). **c**, Three premonocot duplicates of *LPR1*/*LPR*2 remained in *Acorus* (shown in dotted circles). ANA, Amborellales, Nymphaeales and Austrobaileyales.

Because early-branching monocots, including Acorales and Alismatales, are mostly aquatic or wetland plants and show convergent evolution of many diagnostic traits in the aquatic family Hydatellaceae (Nymphaeales), it is believed that ancestral monocots had an aquatic or wetland origin^1^. Intriguingly, expansion of *COG2132* (*LOW PHOSPHATE ROOT1* (*LPR1*) and *LOW PHOSPHATE ROOT2* (*LPR2*)), a group of multicopper oxidases that play a key role in the redox signalling of *Arabidopsis* primary root growth regulated by antagonistic interactions of inorganic phosphate (Pi) and Fe availability^40^ (Fig. 6a), may have played a seminal role in the adaptation of monocots to aquatic habitats with low Pi availability, which is similar to the expansion of *COG2132* in the aquatic eudicot *Nelumbo*^41^ (Fig. 6). We found that aquatic- or wetland-related lineages (*Nymphaea*, *Euryale*, *Acorus*, *Colocasia*, *Nelumbo*, *Oryza*, *Spirodela* and *Zostera*) had higher copy numbers of this gene family than terrestrial plant lineages (two-sided Mann–Whitney *U*-test, *P* < 0.01), which supported the hypothesis that the expansion of *LPR1/LPR2* may have played a seminal role in the aquatic lifestyles of early monocots (Fig. 6b). Whereas five duplication events yielded six copies of *LPR1/LPR2* in *Acorus*, two events occurred before monocot diversification and produced three ancient duplicates, all of which were retained in early-diverged aquatic/wetland monocots, including *Acorus*, seagrass and duckweed (Fig. 6c). These results support the hypothesis that the acquisition of functions drove the aquatic or wetland origin of the monocot ancestor.

## Discussion

Early phylogenetic studies strongly supported *Acorus* as the earliest branching monocot, being sister to all the other extant monocots^7,14^. Our comparative analysis of the *Acorus* genome together with those of other monocots provided further insight into monocot evolution. By identifying only one single palaeopolyploid event during *Acorus* evolution, together with its extremely slow rates of sequence substitution and synteny loss, *Acorus* could be considered a pivotal genome for comparative genomics investigation among monocots (including grasses). Based on this reference, we showed a positive correlation between the synteny loss rate and genome duplication events in monocots and a particularly accelerated evolution rate of genes in the grass family. Polyploidization events are often associated with accelerated rates of species diversification and rapid gene turnover^42^ and adaptation during stressful periods in plants^43^. Within monocots, WGDs are more frequent in cereals (grass family) than in other monocot clades^10,44^, which would give rise to extensive chromosomal rearrangements, karyotype diversification and rapid substitution rates because of relaxed selection on duplicates after a series of WGDs in the grass family^13,31^. This agrees with our result of a positive correlation between the synteny loss rate and genome duplication events in monocots. Because a rapid substitution rate is often a signature of adaptation, whereas rapidly evolving genes also often show neofunctionalization, this rapid rate probably facilitated adaptive radiation of grasses^45^. In addition, in terms of reproductive isolation, according to the reinforcement model of evolution, differentiation of karyotypes often enhances prezygotic isolation and facilitates speciation^46,47^, as we observed in the grass family when compared with other monocots.

With the signature of the slowest evolving lineage, *Acorus* is a good candidate for ancestral monocot genome reconstruction, similar to wax gourd (*Benincasa hispida*) for Cucurbitaceae^48^ and *Amborella trichopoda* for angiosperms^13,15^. This idea was supported by alignments with five representative outgroup taxa which indicated many more ancestral angiosperm genomic regions preserved in *Acorus* than in all the other monocots surveyed. The ancestral genome is often assigned to a hypothesized ‘median’ genome that minimizes the genomic distance between two groups under the DCJ model^49^, such as the ancestral *Brassica* genome^50^ and ancestral legume genome^51^. By including *Acorus* and other early-diverging monocots, we successfully updated the AMK, which further evolved into the five protochromosomes of our previously predicted AMK by two fusions and one fission^13^. Given the lowest rate of synteny loss in *Acorus* among the sequenced monocots when compared with five representative outgroup taxa, these results confirmed the hypothesis that *Acorus* contains the most ancestral genome architecture/karyotype among all the sequenced monocots.

The rhythm of synteny (ancestral gene order) loss via gene deletion and chromosome reshuffling was highly heterogeneous among species and among different functional genes. In high-resolution analyses of genome-wide alignments among monocots and outgroups, we illustrated the complex genome evolutionary patterns during lineage diversification associated with gene-related traits. For example, we observed a negative correlation between higher TE density in syntenic gene-flanking regions and syntenic retention in monocots, which is probably mediated by the movement of TEs. Indeed, mobile elements are normally silenced by epigenetic mechanisms due to their destructive potential. However, they can often be reinvoked in the face of environmental stress and participate widely in chromosomal structural variation as well as genome instability. For example, in *Oryza*, sequence rearrangements are observed more frequently in repetitive regions^52^, which is in line with our results. Moreover, we observed that disrupted synteny during monocot evolution is associated with both the expression level and breadth (inverse of tissue specificity) of a gene. For example, a human–chimpanzee comparative study showed that chromosomal rearrangements, which disrupt synteny, are associated with elevated gene expression differences in the brain^53^. In *Brassica*, homoeologous chromosome rearrangements drive gene expression change in newly resynthesized *Brassica napus* allopolyploids^54^. This could be appropriately addressed by changes in the *cis*-environment of a gene and considerable gene structural mutations, such as unpredictable sequence translocation or inversion when synteny is degraded by complex genetic forces as a whole^55,56^. In a commercial wine yeast strain, an inversion that involves *SSU1* and *GCR1* regulatory regions can activate *SSU1* expression; thus, this inversion facilitates sulphite resistance^57^. Another example in maize shows that an inversion in the *Tu1* mutant with a breakpoint in the promoter of *Zmm19* significantly changes *Zmm19* expression, leading to kernels being completely enclosed in leaflike glumes^58^. Therefore, the genomic position is critical to gene expression. However, co-expressed gene clusters can often be preserved in syntenic blocks in mammals^59^ but not in *Drosophila melanogaster*^60^ or *Arabidopsis*^61^, which probably differ in their constraints on development. However, future studies to test the relationship between co-expression and synteny conservation are needed in different plant species, particularly monocots and cereals. On the other hand, our results also showed that the genes from the outgroup (*Nelumbo*) with higher synteny retention in monocot species exhibit lower nucleotide diversity. This might be attributable to the functional constraints that play an important role in maintaining synteny because rearrangement can have an impact on gene expression^55^ and the abnormal chromosomal pairing and recombination of non-homologous regions can lead to copy number variation or gene loss^62^. Collectively, the gene features observed here shed new light on the intricate evolutionary history of monocot families.

A deep investigation into genome evolution has allowed us to reveal the role of gene copy number variation in specific traits. For example, changes in the MADS-box regulatory gene family related to flower diversity^63^ and massive gene loss in *Cuscuta australis* associated with its parasitic lifestyle^64^ have been reported. In our study, we inferred that substantial gene families probably drove traits associated with the emergence of monocots during flowering plant evolution. Our results displayed a significantly higher copy number of *LPR1/LPR2* in aquatic plants than in terrestrial plants, which is consistent with previous findings in *Nelumbo*^41^. The expansion of *LPR1/LPR2* is believed to be associated with a low-phosphate aquatic environment, especially in low Pi conditions^41^. Phosphorus (P) is one of the major nutrient limitations in many freshwater ecosystems, including streams and wetlands^65^. In *Arabidopsis, LPR1* and its homologue *LPR2* regulate root meristem activity related to Pi availability^66,67^. Although low Pi can inhibit primary root growth in wild-type *Arabidopsis*, increasing the gene products of *LPR1* by overexpression can further inhibit primary root growth under low Pi conditions; by contrast, the loss-of-function *lpr1lpr2* double-mutant showed enhanced primary root growth under low Pi conditions^40^. In *Nelumbo*, the increased copies of *LPR1/LPR2* were found to be highly expressed in its lateral and adventitious root primordia^41^. All these results probably suggest a shared evo–devo strategy in both early-branching aquatic monocots and other aquatic angiosperms to form ephemeral primary roots instead of taproots in response to low Pi in streams or wetlands^68^. Moreover, by utilizing lateral spreading, together with the development of adventitious roots, early monocots can adapt to wetland habitats with differential moisture contents close to that of the Earth’s surface^68^. Apart from *LPR1*/*LPR2*, we also found that losses of the non-monocot-conserved *DOT3* gene in monocots were linked not only to the emergence of ephemeral primary roots, but also to parallel venation in these clades^39^. Finally, we revealed that *WOX1*, an essential gene that regulates cotyledonary primordia initiation^69,70^, is completely lost in monocots, suggesting that an ancient loss occurred before modern monocots diverged. Such loss is very probably attributed to the formation of the single cotyledon character that is unique to monocots, which still needs more studies to be further investigated.

## Methods

### Plant material, genome sequencing and RNA-seq of *Acorus*

*Acorus tatarinowii* (NCBI Taxonomy ID: 123564) was collected from Shennongjia Nature Reserve (Hubei, China). DNA from leaves was extracted using Plant DNA Isolation Reagent (TIANGEN). For genome size estimation, genomic DNA was sheared into 250–280 bp fragments with the NEBNext Ultra DNA Library Prep Kit for Illumina (NEB) based on the manufacturer’s protocol. Paired-end reads (150 bp for each end) were sequenced on the Illumina HiSeq 4000 platform. DNA libraries were constructed based on the PacBio library preparation protocol and further sequenced on the PacBio Sequel platform (Pacific Biosciences) with the Sequel II Binding Kit 1.0, Sequel II Sequencing Kit 1.0 and Sequel II SMRT Cell 8M at Frasergen. Subread data was obtained via SMRT LINK 7.0. Subreads with a quality score below 0.8 were excluded. The Hi-C DNA library was prepared at Frasergen using a previously published protocol^71^. Generally, nuclear DNA was cross-linked inside tissue cell samples of young leaves. The extracted DNA was further digested using the restriction enzyme MboI. Biotinylation was tagged at both sticky ends of the digested DNA fragments and then ligated randomly after dilution. The condensed, sheared and biotinylated DNA fragment libraries were prepared for paired-end sequencing with a 150-bp read length on an Illumina HiSeq platform. For transcriptome sequencing, total RNA of young leaves, old leaves and roots was extracted using the RNAprep Pure Plant Kit (TIANGEN). Quality checking was performed on 1% agarose gels, and the RNA concentration and integrity were further assessed by a Qubit RNA Assay Kit in a Qubit 2.0 Fluorometer (Life Technologies) and Agilent 2100 Bioanalyzer (Agilent Technologies), respectively. Qualified RNAs of each sample (3 μg) were then used to construct the Illumina sequencing library according to the recommendations of the NEBNext Ultra RNA Library Prep Kit for Illumina (NEB). The libraries were sequenced on the Illumina HiSeq 2500 platform at Novogene with 150 bp paired-end reads.

### Chromosomal-level assembly of *Acorus tatarinowii*

The genome size and heterozygosity of *Acorus* were estimated by jellyfish^72^ and genomic charactor estimator^73^ using *k*-mer frequency distribution (*k*-mer = 17 as the default) based on Illumina reads with default settings. For genome assembly, Nextdenovo software v.2.5.0 was applied for PacBio read correction with the following parameters: read_cutoff = 1k; seed_cutoff = 40,150; blocksize = 1g; pa_correction = 2; seed_cutfiles = 2; sort_options = -m 4g -t 10 -k 50; minimap2_options_raw = -x ava-pb -t 8; correction_options = -p 15. The corrected PacBio reads were further trimmed and assembled by Canu v.2.2 with the trimming parameters ‘genomeSize = 400m; correctedErrorRate = 0.12; corMaxEvidenceErate = 0.15; minReadLength = 1,000; minOverlapLength = 500; merylThreads = 40’ and the assembling parameters ‘genomeSize = 400m; maxThreads = 60; correctedErrorRate = 0.035’ (https://github.com/Nextomics/NextDenovo). After mapping PacBio reads on the polished contigs, redundant contigs were removed by purge_haplotigs based on read coverage. The Hi-C sequencing reads were aligned to the final contigs by BWA-MEM^74^. Finally, scaffolding of these contigs into pseudochromosomes was performed with LACHESIS^75^. Juicer was applied to construct high-resolution contact maps of chromosomes, and JuiceBox v.2.1.10 was further used to visually correct the assembly errors, including the orientation, order and internal misassembly of contigs^76^.

### Repeat, gene and functional annotations

Before gene annotation, repeat sequences including TEs on the chromosome-level assembly were de novo predicted using Extensive de novo TE Annotator (EDTA, v.1.8.4) with default settings^77^ and annotated by RepeatMasker (http://www.repeatmasker.org). Genes were predicted by combining: (1) RNA-seq evidence, (2) protein homology and (3) ab initio prediction. For gene prediction with transcriptional evidence, RNA-seq reads from our newly sequenced young leaf, old leaf, root and publicly available rhizome and leaf data (accession no. SRR9644796 and SRR9644797) were aligned to the assembly by the HISAT2-StringTie pipeline to obtain transcript-based annotation^78,79^. CDSs were predicted using Transdecoder (https://github.com/TransDecoder). In addition, the de novo transcriptome was assembled by Trinity with default settings (https://github.com/trinityrnaseq/trinityrnaseq); PASA, which integrated the de novo transcript assemblies, was applied to further update the assembly with default settings (https://github.com/PASApipeline/PASApipeline). Homology-based gene annotation was conducted using Genewise software with genomic sequences and gene annotations from representative monocots, including *Colocasia esculenta* (accession no. ASM944546v1), *Zea mays* (no. B73 RefGen_v4), *Oryza sativa* (no. GCF_000005425) and *Zostera marina* (no. GCA_001185155.1)^80^. Ab initio gene prediction was conducted using AUGUSTUS^81^ and GeneMark-ES/ET^82^. The final consensus gene annotations were generated by EVidenceModeler with different weights among annotations (RNA-seq > gene homology > ab initio)^83^. Finally, protein-coding genes with more than 30% of the CDS overlapping with repeat sequences were considered repeat- or transposon-related genes and were discarded from downstream analyses. GO functional annotations were inferred using the ‘non-redundant’ database of plants in eggNOG 4.5 with default settings^84^.

### AMK reconstruction

Ancestral genomes are reconstructed in a six-step method as illustrated in Fig. 4a. The first step consists of aligning the genes (protein sequences) using BlastP with thresholds for cumulative identity percentage (CIP) ≥50% and cumulative alignment length percentage BLAST parameters (CALP) ≥50% (defined in Salse et al.^85^) (https://github.com/nelumbolutea/amk_article/blob/main/6.CIP_CALP.pl), which deliver conserved genes between the investigated species given the following formulas:

$${{{\mathrm{CIP}}}} = {\sum} {{{{\mathrm{nb}}}}\;{{{\mathrm{ID}}}}\;{{{\mathrm{by}}}}\;{{{\mathrm{(HSP/AL) \times 100}}}}}$$

where CIP corresponds to the cumulative percentage of sequence identity observed for all the high-scoring pairs (HSPs) divided by the cumulative aligned length (AL) which corresponds to the sum of all HSP lengths. The ‘nb’ denotes number.

$${{{\mathrm{CALP = }}}}\frac{{{{{\mathrm{AL}}}}}}{{{{{\mathrm{Query}}}}\;{{{\mathrm{length}}}}}}$$

where CALP is the sum of the HSP lengths (AL) for all HSPs divided by the length of the query sequence. With these parameters, BLAST produces the highest cumulative percentage identity over the longest cumulative length, thereby increasing stringency in defining conserved genes between two genome sequences^85^. The second step consists of removing species-specific and local (tandem) duplicates and retaining only the single-copy orthologues, which will reveal that protogenes conserved in all the investigated species or between a subset (at least two) of the investigated species. This step consists in extracting one-to-one gene relationships between species from the step 1 output file. The third step consists of clustering or chaining groups of conserved genes into synteny blocks (SBs). The third step consists of extracting all combinations of chromosome-to-chromosome relationships (for SBs sharing more than five orthologous genes) from the step 2 output file (or alternatively using tools such as DRIMM synteny software^86^). In the fourth step, SBs from the previous output file are then merged into ancestral protochromosomes (also referred to as CARs). This step consists of defining independent groups of SBs sharing synteny between the modern species investigated (or alternatively with tools such as MGRA software^87^ or ANGES software^88^). The fifth step corresponds to CAR validation, in which CARs correspond exclusively to diagonals in dotplot-based comparative genomics deconvolutions of the synteny between the investigated species. Finally, the sixth step consists of deriving a parsimonious evolution model by introducing the smallest number of rearrangements (fissions, fusions and translocations) to explain the transition between the ancestral and modern genomes. This strategy has been previously applied to infer a pre-τ AMK structured into 5 protochromosomes with 6,707 genes (available in Supplementary Table 3 from Murat et al.^13^) at the MRCA of *Ananas* (pineapple), *Elaeis* (palm) and grasses. In the current study, we use this *n* = 5 AMK as a pivot to compare, in a BlastP and dotplot-based approach, the modern karyotypic structures of the *Acorus* genome and other early-branching monocot genomes, including *Spirodela polyrhiza*, *Colocasia esculenta* and *Dioscorea* (*alata* and *rotundata*). From the gene (protein sequences) alignments using BlastP, and CIP and CALP parameters of the pre-τ AMK compared with *Acorus*, *Spirodela*, *Colocasia* and *Dioscorea*, stored in a tabular format to further extract from it conserved genes (step 1), one-to-one gene orthologous relationships (step 2), SBs (step 3) and CARs (step 4), as well as dotplot illustrations of the synteny between the investigated species, we proposed the karyotypic structures of the ancestral monocots (Step 5) and inferred an evolutionary scenario taking into account the fewest number of genomic rearrangements (including inversions, deletions, fusions, fissions and translocations) that may have occurred between the AMK and modern monocot genomes. All data described in the current study, such as conserved genes, SBs and ancestral chromosome blocks, are available in Supplementary Tables 5 and 6.

### Gene and WGD analyses

To identify the origins of genes from duplications and WGDs in *Acorus*, intraspecific and interspecific SBs were identified by MCscan via JCVI^89^. To determine the WGDs in relation to the divergence of rice, asparagus and seagrass, raw 4dTv values for all syntenic paralogous pairs or orthologous pairs were estimated and corrected for possible multiple transversions at the same site according to a previous method^90^; *K*_S_ values of all syntenic paralogous/orthologous pairs were also calculated by codeML of the PAML package^91^. Histograms of 4dTv and *K*_S_ values for all syntenic paralogues/orthologues were plotted with a bin size of 0.01. Subgenome fractionation analysis of *Acorus* was performed as outlined previously^92^. The numbers of collinear genes (ancestral genes) and non-collinear genes were counted for pairs of syntenic blocks and tested for significant fractionation bias (χ^2^ test). Collinear genes refer to those *Acorus* genes showing syntenic relationships to any of the remaining 42 monocots (including *Acorus*) (Supplementary Table 13), whereas non-collinear genes are those without synteny to any monocot species considered. LF and MF syntenic blocks were assigned based on differences in the numbers of collinear genes. To better validate and visualize LF and MF blocks, we calculated syntenic gene retention of *Acorus* LF and MF blocks in six representative outgroups, *Amborella trichopoda*, *Aristolochia fimbriata, Spirodela polyrhiza*, *Elaeis guineensis*, *Nelumbo nucifera* and *Aquilegia coerulea*. TE sequence proportions between collinear genes in LF and MF syntenic blocks were compared with sliding windows in gene-flanking regions (±5 kb) and gene bodies (from the translation start site to the stop site). Any genomic positions overlapping between the flanking region and gene were discarded during analysis of the flanking regions. For both flanking regions, a 100-bp sliding window with a 10-bp step was used, whereas 40 evenly divided windows were applied for genes^93^. Furthermore, for each sliding window, the proportion of the sequence belonging to TEs was summarized. The average proportion in each sliding window was calculated for genes in LFs and MFs. These averaged proportions represent the TE density in the flanking regions and genes in LFs and MFs. Moreover, to investigate subgenome dominance (biased expression levels between LFs and MFs)^94^, all five *Acorus* RNA-seq datasets used for gene annotation were surveyed. Gene expression levels (FPKMs) were calculated by HISAT2-StringTie pipeline^60,61^. For each RNA-seq dataset, log_2_-transformed FPKM values for anchor genes from LFs and MFs were compared using the one-sided paired *t* tests in GraphPad Prism v.9.

### Sequence substitutions and synteny retention among monocots

To compare the relative sequence substitutions among monocots, we surveyed 42 monocots with available genome assemblies, including *Acorus*, and six outgroup taxa (Fig. 2a and Supplementary Table 13). The species tree was constructed based on 104 strict single-copy orthologous genes using OrthoFinder^95^. We concatenated single-copy genes and generated a phylogenetic tree by IQ-TREE2 under the optimal substitution model JTT + F + I + G4 according to the Bayesian information criterion scores of 144 tested models^96^. The relative substitution rate of each monocot is the sum of all branch lengths from the taxon tip to the node of the MRCA of monocots in the phylogenetic tree. To estimate the variation in the synteny loss rate among monocots, monocot genomes were aligned to outgroup taxa, including *Amborella trichopoda* (the earliest branching angiosperm) (CoGe id50948)^15^, *Nymphaea colorata* (Nymphaeales)^20^, *Aristolochia fimbriata* (a Magnoliidae species without a WGD)^21^, *Cinnamomum kanehirae* (magnoliids)^22^ and *Nelumbo nucifera* (eudicot)^24^, by McScan via JCVI^89^. The size of a syntenic block was represented by the number of anchor gene pairs in the block, whereas the relative synteny retention rate was represented by the total number of genes in an outgroup taxon with a syntenic relationship to a monocot. Furthermore, Pearson correlations between synteny retention rates and key factors (expected number of gene copies after ancient WGDs, genome sizes, substitution rates) were calculated. The number of ancient WGDs in each monocot was inferred from published literature (Supplementary Table 13).

### Synteny retention among different genes

To estimate the variation in synteny retention among different genes during monocot radiation, we used the outgroup taxon *Nelumbo nucifera* because of its greatest similarity of syntenic structure in relation to monocots, and the availability of required datasets including whole-genome methylation, population resequencing and expression profiles of all organs and tissues^12,24^. The 29,582 *Nelumbo* genes sharing homologue(s) (BlastP *E* value <10^−6^) with at least 1 of the 42 monocots were used for the following analysis of synteny retention rates. For each *Nelumbo nucifera* gene, the number of monocots showing a syntenic relationship was used to represent its relative synteny retention rate during monocot radiation. To gain insight into different factors related to synteny retention rates among genes, data including the types of gene duplications, nucleotide diversity, CDS length, gene length, the number of predicted PPIs, the average expression level, expression specificity, TE density and methylation levels on genes and flanking regions were obtained from our previous study^24^. Among the types of gene duplications, WGD genes (genes retained from WGD), tandem duplicates (tandemly duplicated genes), single-copy genes (genes without homologues within *Nelumbo*), proximal duplicates (duplicated having one or a few intervening genes) and WGD&tandem duplicates (genes that underwent both WGD and tandem duplications) were classified using MCscanX in our previous study^24^. While the two-sided Mann–Whitney *U-*test was applied to compare retention rates among genes from different types of duplications (WGDs, tandem, proximal, single-copy and dispersed), Pearson correlations were calculated between synteny retention rates and different factors, such as *π* and CDS length, for all *Nelumbo nucifera* genes using R (https://www.r-project.org/). Meanwhile, *Nelumbo* genes sharing homologue(s) with at least one monocot were further divided into four groups following a decreasing number of monocots with synteny retention. Levels of each gene trait among groups I, II, III and IV were compared using the Kruskal–Wallis test in GraphPad Prism v.9.

### Evolution of functional genes at the emergence of monocots

To gain insight into OG evolution in the ancestral monocot, 28 representative taxa, including early-branching angiosperms, monocots and eudicots, were used for comparisons. First, OGs were obtained via OrthoFinder^95^. Single-copy genes identified from OGs were aligned using protein sequences by MAFFT, and a species tree was built based on concatenated single-copy gene alignment using IQTREE with the parameters described above. The species tree rooted with *Ginko* was used as an input to build an ultrametric tree (chronogram) by r8s, whereas fossil constraints were set to *Arabidopsis–Nymphaea* (125–247.2 Ma), *Arabidopsis*–*Liriodendron* (125–247.2 Ma), *Arabidopsis*–*Oryza* (125–247.2 Ma) and *Arabidopsis*–*Aquilegia* (−128.63 Ma) according to a previous study^20^. To estimate OG gain and loss along the ultrametric tree, we applied Dollo-Parsimony via COUNT software with default settings^97^. To estimate the number of OGs with significant expansion and contraction along the ultrametric tree, CAFE was applied with a *P* value threshold of 0.05 (ref. ^98^). In parallel, to better detect OGs with significant copy number differences between monocots and non-monocot angiosperms, the copy numbers of these two clades were compared using the two-sided Mann–Whitney *U-*test for each OG. OGs with a *P* value <0.01 and fold change of the average copy number ≥2 were considered significantly different.

### Reporting summary

Further information on research design is available in the Nature Research Reporting Summary linked to this article.

## Supplementary information

Supplementary Information Supplementary Figs. 1–15.

Reporting Summary

Supplementary Tables Supplementary Tables 1–13.

## Data Availability

The datasets generated and analysed during the current study including PacBio Sequel II, Illumina, Hi-C data, genome assembly, annotation and RNA-seq reads have been deposited in China National GeneBank (CNGB, https://db.cngb.org/) under accession number CNP0001708. Public transcriptomes used in this study are available from NCBI under the accession number SRR9644796 and SRR9644797. Source data are provided with this paper.

## Source data

Source Data Fig. 1 Statistical source data.
Source Data Fig. 2 Statistical source data.
Source Data Fig. 3 Statistical source data.
Source Data Fig. 5 Statistical source data.
Source Data Extended Data Fig. 2 Statistical source data.
Source Data Extended Data Fig. 3 Statistical source data.
Source Data Extended Data Fig. 4 Statistical source data.
Source Data Extended Data Fig. 5 Statistical source data.

## Code availability

The main custom scripts and workflow have been deposited in Github (https://github.com/nelumbolutea/amk_article).
