# Supplementary material for: The slow-evolving Acorus tatarinowii genome sheds light on ancestral monocot evolution
Source: Nat Plants. 2022 Jul 14;8(7):764–77. doi: 10.1038/s41477-022-01187-x (PMC9300462; doi:10.1038/s41477-022-01187-x)
Supplement: Supplementary file 1 — Supplementary Figs. 1–15. [file 41477_2022_1187_MOESM1_ESM.pdf]

nature portfolio

https://doi.org/10.1038/s41477-022-01187-x

Supplementary information

The slow-evolving *Acorus tatarinowii* genome sheds light on ancestral monocot evolution

In the format provided by the authors and unedited

The slow-evolving *Acorus tatarinowii* genome sheds light on ancestral monocot evolution

Tao Shi[1,2], Cécile Huneau[3], Yue Zhang[1,2,4], Yan Li[1,2,4], Jinming Chen[1,2*], Jérôme Salse[3*], Qingfeng Wang[1,2,5*]

[1] CAS Key Laboratory of Aquatic Botany and Watershed Ecology, Wuhan Botanical Garden, Chinese Academy of Sciences, Wuhan, China

[2] Center of Conservation Biology, Core Botanical Gardens, Chinese Academy of Sciences, Wuhan, China

[3] INRA-UCA UMR 1095 Génétique Diversité et Ecophysiologie des Céréales, 63100, Clermont-Ferrand, France

[4] University of Chinese Academy of Sciences, Beijing, China

[5] Sino-African Joint Research Center, Chinese Academy of Sciences, Wuhan, China

[*]Corresponding author E-mails: jmchen@wbgcas.cn; jerome.salse@inrae.fr; qfwang@wbgcas.cn

Supplementary Figures

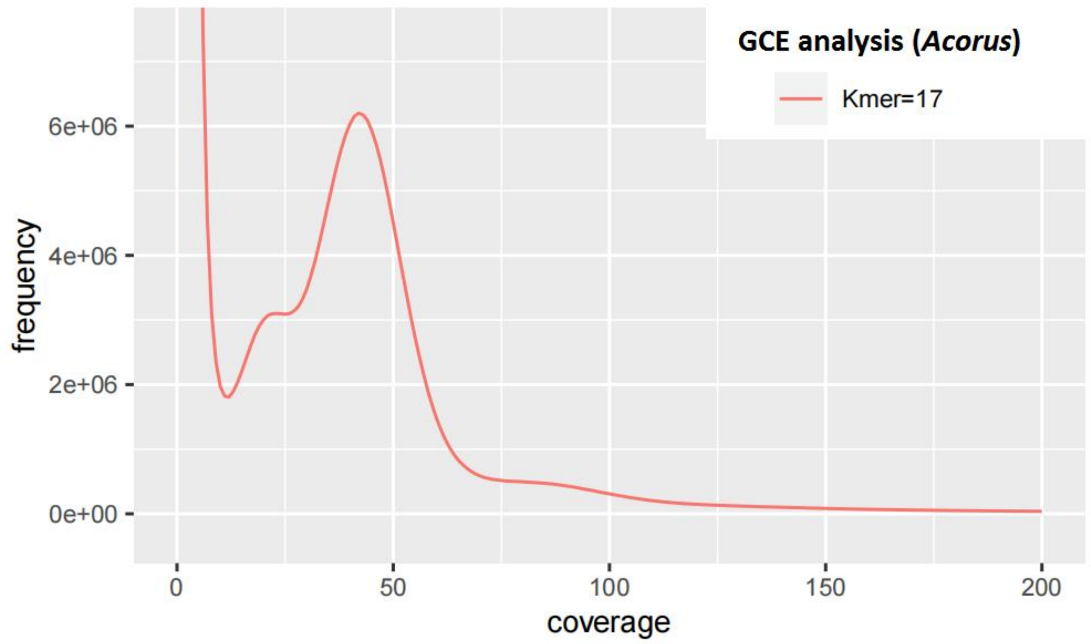

Supplementary Figure S1. K-mer (k = 17) analysis for estimating the genome size of *Acorus based* on Illumina reads. X-axis is the depth of read coverage. Y-axis is the frequency at that depth divided by the total frequency of all depths. Genome size = K-mer number /peak depth. The secondary peak at a position half of that of the main peak represents the rate of heterozygosity.

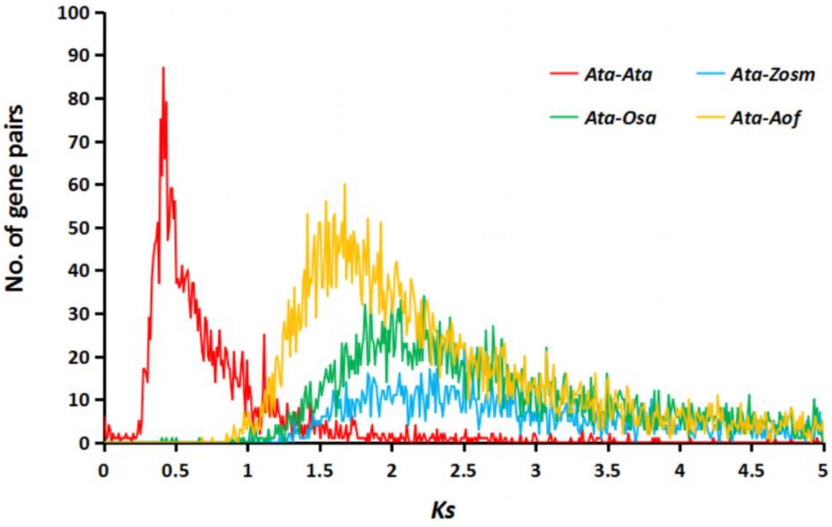

Supplementary Figure S2. **Density distributions of syntenic paralogs of *Acorus* and syntenic orthologs between Acorus and other monocots according to Ks divergence.** Ata: Acorus; Zosm: seagrass; Osa: rice; Aof: asparagus.

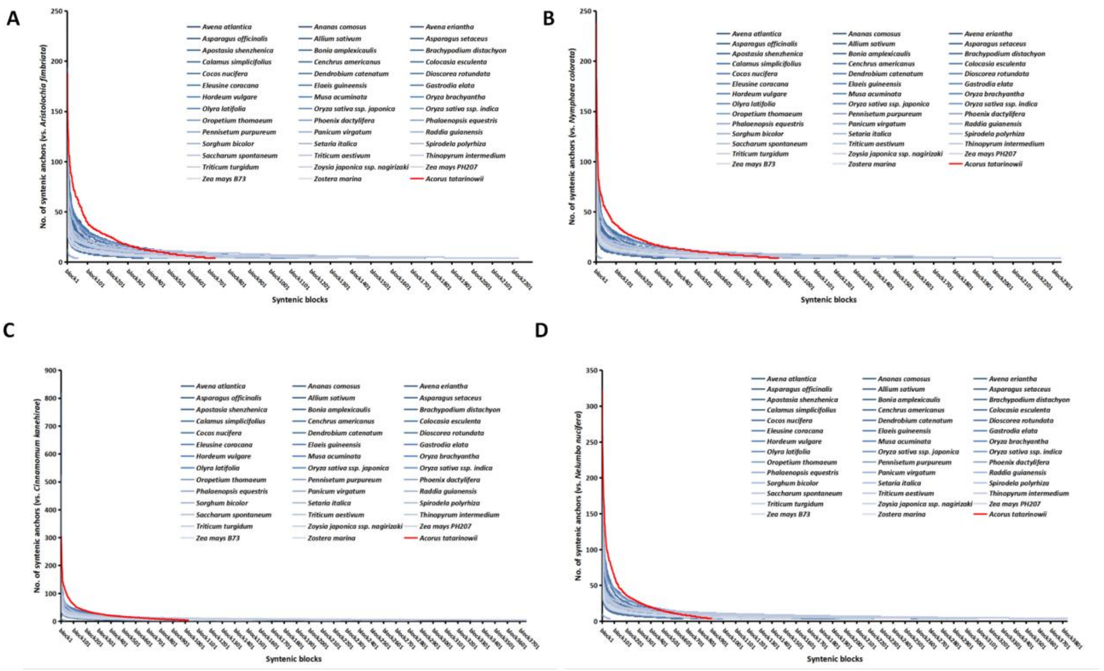

Supplementary Figure S3. Comparison of 'syntenic block size' decay among different monocot species when compared to the outgroups *Aristolochia* (A), *Nymphaea* (B), *Cinnamomum* (C) and *Nelumbo* (D).

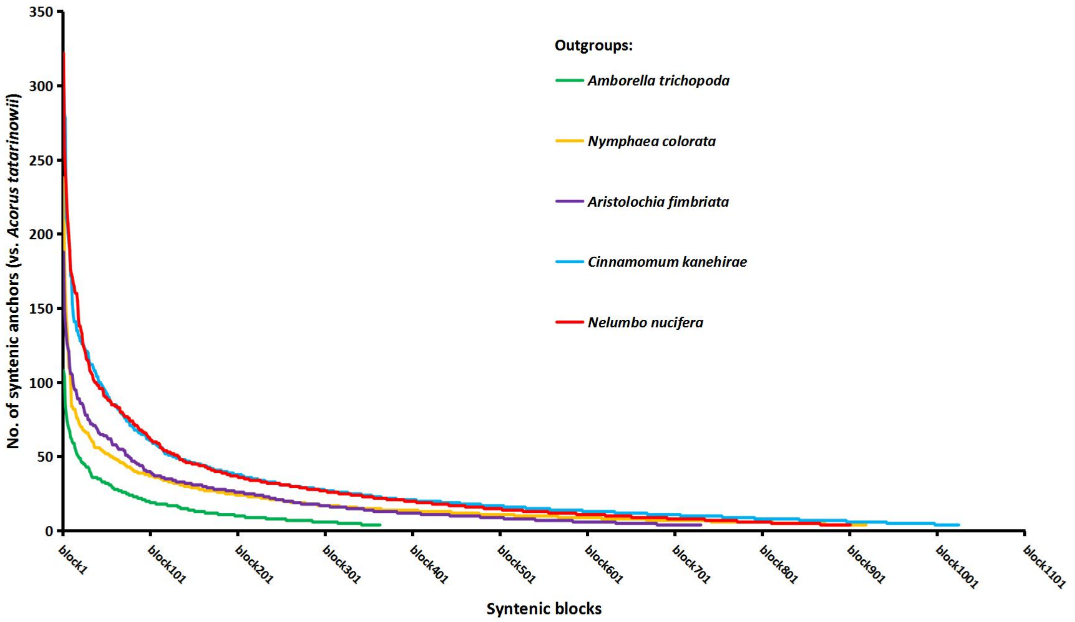

Supplementary Figure S4. Comparison of 'syntenic block size' decay among different outgroup species (*Amborella*, *Nymphaea*, *Aristolochia, Cinnamomum* and *Nelumbo*) when compared to *Acorus*.

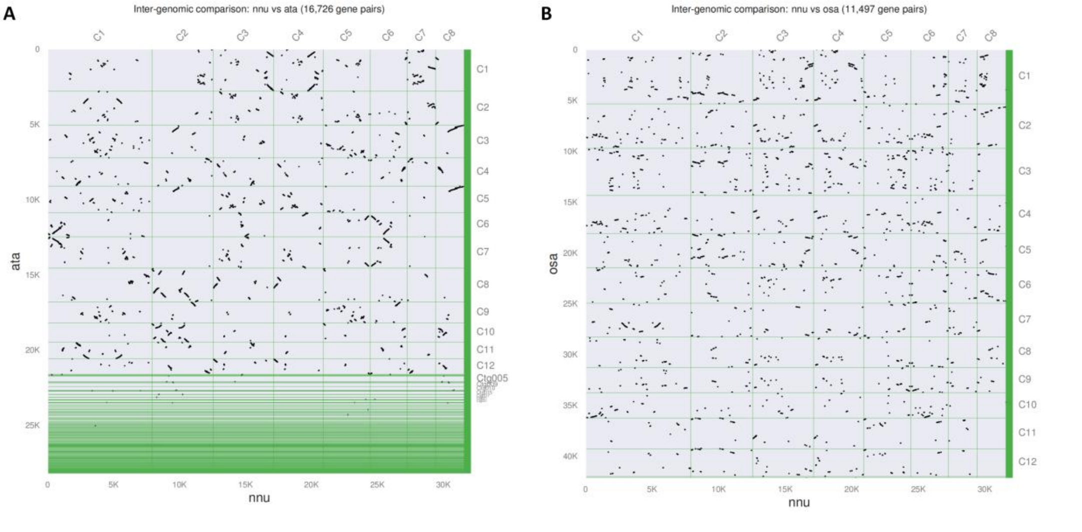

Supplementary Figure S5. **Scatter plot of between-species syntenies. A**. Synteny between *N. nucifera* (nnu) and *Acorus* (ata). **B**. Synteny between *N. nucifera* (nnu) and rice (osa).

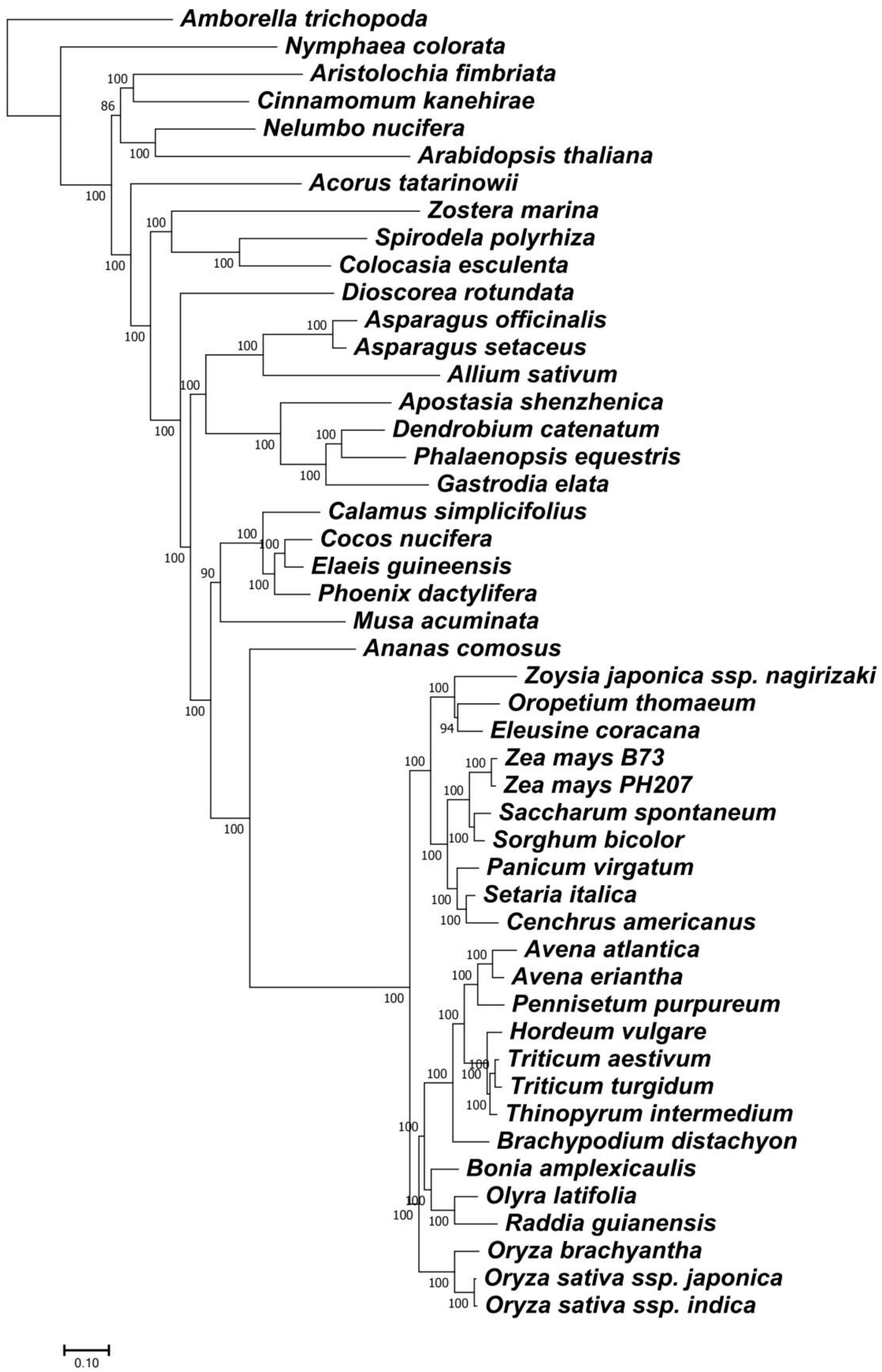

Supplementary Figure S6. **Phylogenetic tree of monocots and outgroups based on concatenated single-copy gene alignments.** Branch length: number of amino acid substitutions per site. Nodes: bootstraps.

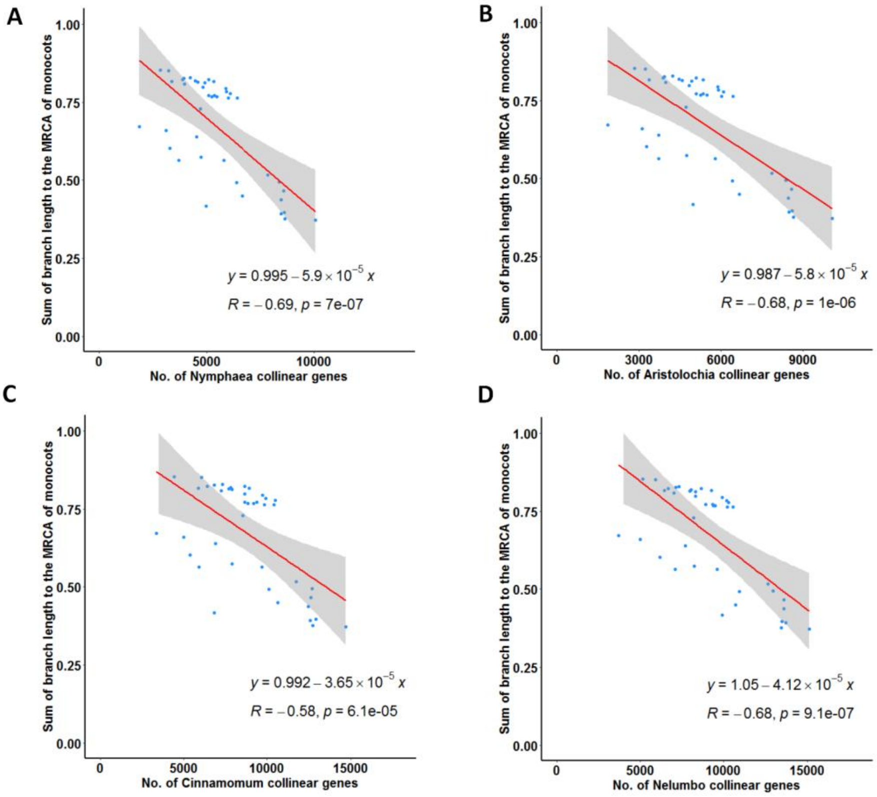

Supplementary Figure S7. Significantly negative correlation (calculated by Pearson's correlation) between the number of syntenic genes in *Nymphaea* (A)/ *Aristolochia* (B)/ *Cinnamomum* (C)/ *Nelumbo* (D) and the sum of branch length to the MRCA of monocots in the concatenated single-copy gene tree. The error bands represent 95% confidence intervals based on a binomial model.

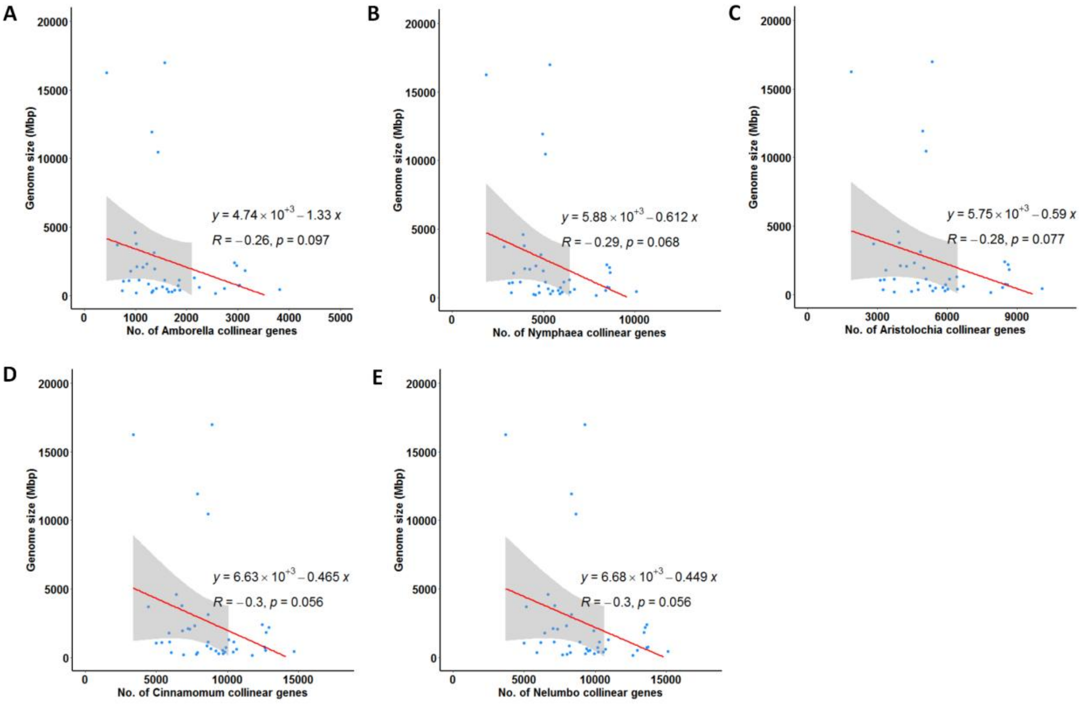

Supplementary Figure S8. **No significantly negative correlation (calculated by Pearson's correlation) between the number of syntenic genes in *Amborella* (A)/ *Nymphaea* (B)/ *Aristolochia* (C)/ *Cinnamomum* (D) and *Nelumbo* (E) genome size. The error bands represent 95% confidence intervals based on a binomial model.**

AMK Shi et al 2022	AMK Murat et al 2027	AMK Xu et al 2021
1	chr1(454)	chr1(4),chr2(5),chr4(4),chr5(144),chr6(5),chr7(3)
2	chr2(885)	chr1(7),chr2(190),chr3(13),chr4(2),chr5(6),chr6(37),chr7(12)
3	chr3(982)	chr1(13),chr2(7),chr3(3),chr4(280),chr5(4),chr6(2),chr7(3)
4	chr4(424)	chr1(10),chr2(2),chr3(92),chr4(17),chr5(3),chr6(3),chr7(4)
5	chr5(427)	chr1(3),chr2(1),chr3(1),chr4(3),chr5(2),chr6(121),chr7(7)
6	chr1(307),chr5(86)	chr1(1),chr2(1),chr3(1),chr4(5),chr5(82),chr6(2),chr7(18)

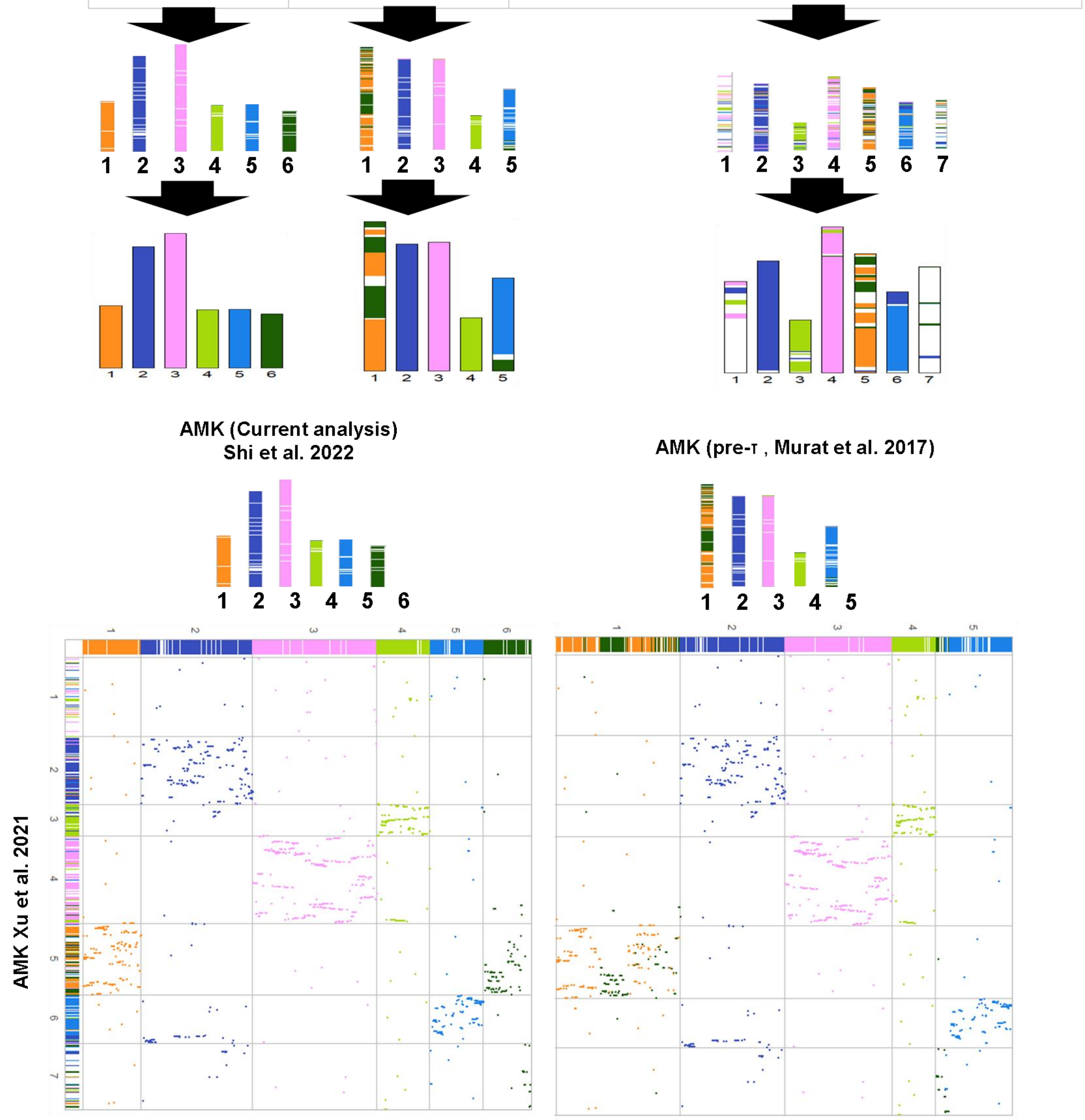

Supplementary Figure S9. **Comparison of the structures of the current n=5 AMK to the n=6 AMK from Murat et al. (2017) and n=7 AMK from Xu et al. (2021).** The

chromosome-to-chromosome relationships between the current n=6 AMK (Shi et al. 2022), n=5 AMK from Murat et al. (2017) and n=7 AMK from Xu et al. (2021), with the orthologous chromosomes highlighted in red for the n=7 AMK from Xu et al. (2021), defining the following chromosome correspondence of 1-1-5, 3-3-4, 5-5-6, 2-2-(2-6), 4-4-(3-4), 6-(1-5)-(5-7), respectively in the current n=6 AMK, n=5 AMK from Murat et al. (2017) and n=7 AMK from Xu et al. (2021). Dotplot-based comparison of the chromosomal relationship between the current n=5 AMK and n=6 AMK from Murat et al. (2017), in x-axis, and the n=7 AMK from Xu et al. (2021), in y-axis illustrating the previous chromosome synteny relationships between the proposed ancestors.

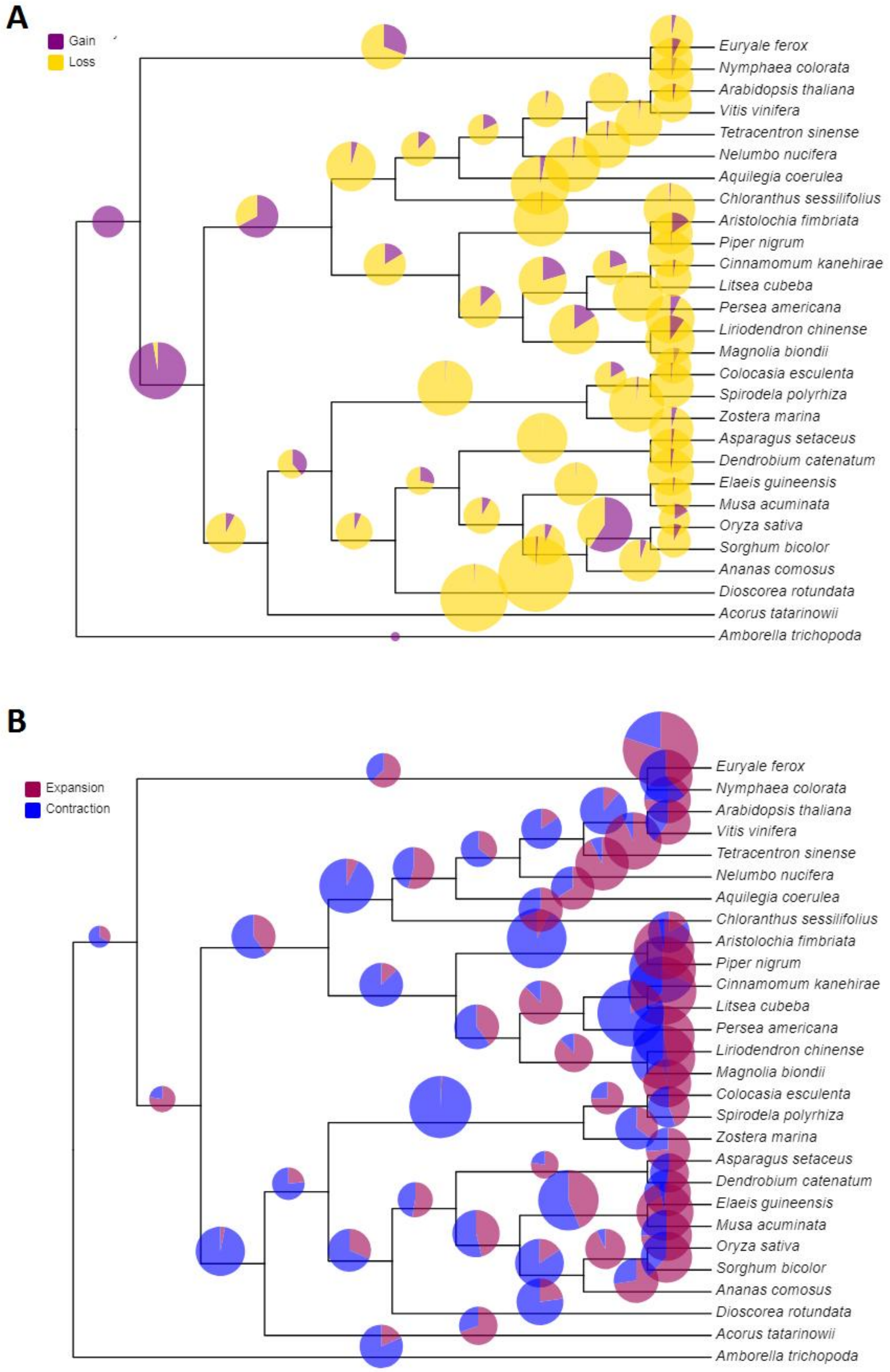

Supplementary Figure S10. **Ortholog group (OG) evolution of representative plants.** A. OG gain and loss based on Dollo parsimony. B. Significant expansion and contraction of OGs by CAFÉ analysis . Only OGs with at least two members are analyzed.

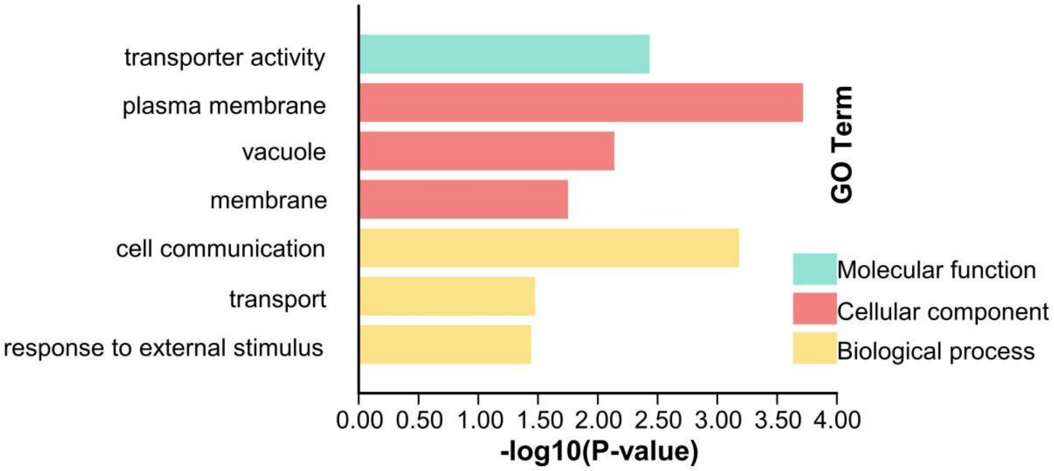

Supplementary Figure S11. **Significantly enriched Gene Ontology (GO) terms for OGs gained in the ancestral monocot. P-values were obtained using an one-sided hypergeometric test.**

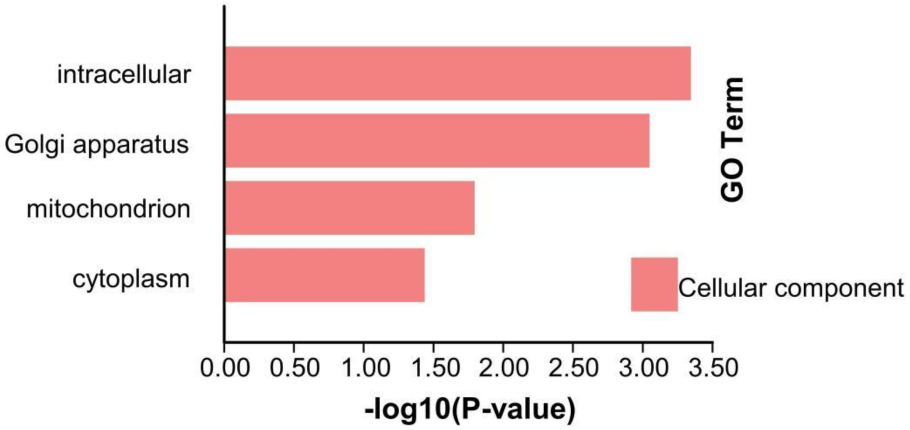

Supplementary Figure S12. Significantly enriched Gene Ontology (GO) terms for OGs lost in ancestral monocot. P-values were obtained using an one-sided hypergeometric test.

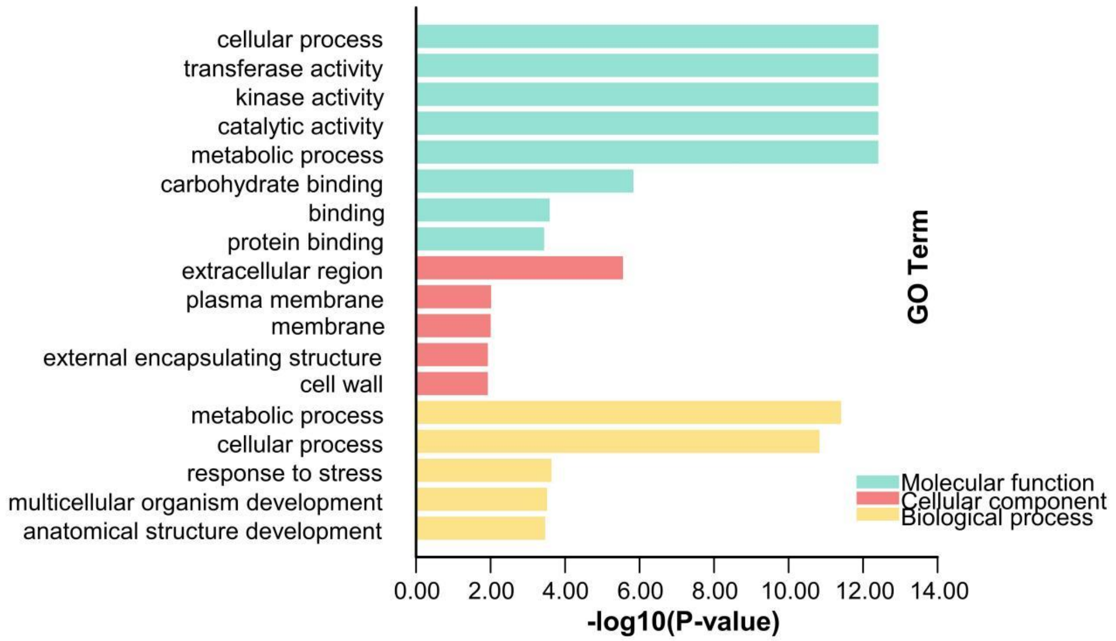

Supplementary Figure S13. Significantly enriched Gene Ontology (GO) terms for OGs expanded in ancestral monocot by CAFÉ analysis. P-values were obtained using an one-sided hypergeometric test.

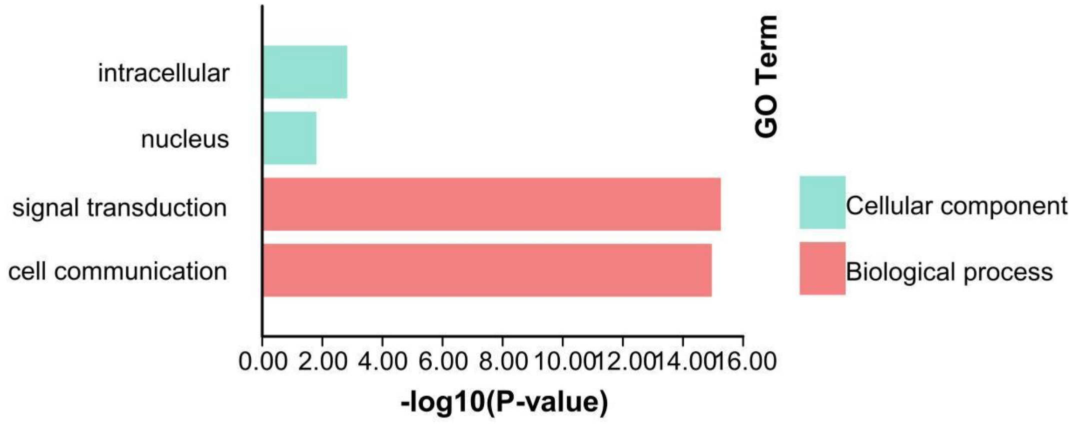

Supplementary Figure S14. Significantly enriched Gene Ontology (GO) terms for OGs contracted in ancestral monocot by CAFÉ analysis. P-values were obtained using an one-sided hypergeometric test.

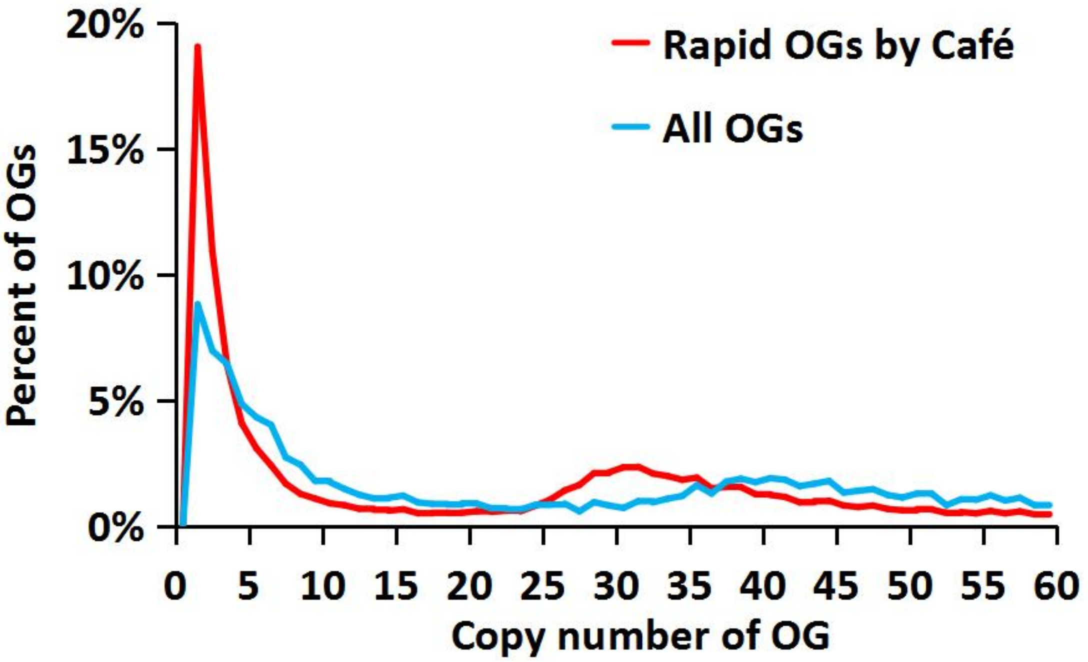

Supplementary Figure S15. Density distribution of all ortholog groups (OGs) and rapidly evolving OGs by CAFÉ analysis according to an incremental OG size.
