# Supplementary material for: The slow-evolving Acorus tatarinowii genome sheds light on ancestral monocot evolution
Source: Nat Plants. 2022 Jul 14;8(7):764–77. doi: 10.1038/s41477-022-01187-x (PMC9300462; doi:10.1038/s41477-022-01187-x)
Supplement: Supplementary file 2 — Reporting Summary [file 41477_2022_1187_MOESM2_ESM.pdf]

nature portfolio

Corresponding author(s): Jinming Chen, Jérôme Salse, Qingfeng Wang

Last updated by author(s): Apr 21, 2022

Reporting Summary

Nature Portfolio wishes to improve the reproducibility of the work that we publish. This form provides structure for consistency and transparency in reporting. For further information on Nature Portfolio policies, see our Editorial Policies and the Editorial Policy Checklist.

Statistics

For all statistical analyses, confirm that the following items are present in the figure legend, table legend, main text, or Methods section.

n/a	Confirmed	
☐	☒	The exact sample size (*n*) for each experimental group/condition, given as a discrete number and unit of measurement
☐	☒	A statement on whether measurements were taken from distinct samples or whether the same sample was measured repeatedly
☐	☒	The statistical test(s) used AND whether they are one- or two-sided<br>*Only common tests should be described solely by name; describe more complex techniques in the Methods section.*
☒	☐	A description of all covariates tested
☐	☒	A description of any assumptions or corrections, such as tests of normality and adjustment for multiple comparisons
☐	☒	A full description of the statistical parameters including central tendency (e.g. means) or other basic estimates (e.g. regression coefficient) AND variation (e.g. standard deviation) or associated estimates of uncertainty (e.g. confidence intervals)
☐	☒	For null hypothesis testing, the test statistic (e.g. F, t, r) with confidence intervals, effect sizes, degrees of freedom and P value noted<br>*Give P values as exact values whenever suitable.*
☒	☐	For Bayesian analysis, information on the choice of priors and Markov chain Monte Carlo settings
☒	☐	For hierarchical and complex designs, identification of the appropriate level for tests and full reporting of outcomes
☐	☒	Estimates of effect sizes (e.g. Cohen's *d*, Pearson's *r*), indicating how they were calculated

Our web collection on statistics for biologists contains articles on many of the points above.

Software and code

Policy information about availability of computer code

Data collection	No software of collecting the data.
Data analysis	For Plant Material, PacBio Sequel, HI-C Sequencing and RNA-seq of Acorus tatarinowii<br>SMRT LINK v7.0<br><br>For genome size estimation:<br>Jellyfish v2.3.0<br>GCE v1.0.2<br><br>For Chromosomal-level assembly of Acorus tatarinowii<br>Nextdenovo v2.5.0<br>Canu v2.2<br>BWA v0.7.17<br>LACHESIS v2017-12-21<br>JucieBox v2.1.10.<br><br>For Repeat, gene and functional annotations<br>EDTA v1.8.4<br>RepeatMasker v4.1.2<br>HISAT2 v2.2.1<br>StringTie v2.1.5<br>Trinity v2.13.2<br>PASA v2.3.1

Genewise v2.4.0
AUGUSTUS v2.5.5
GeneMark-ES/ET v4.68
eggNOG v4.5

For Gene and whole-genome duplication analyses
JCVI v1.1.12
PAML v4.8
BLAST+ 2.12.0
Graphpad PRISM v9.0.1
OrthoFinder v2.3.11
MAFFT v7.49
IQTREE v1.6.12
r8s v1.9
COUNT v9.1106
CAFE v4.1.1

For ancestral karyotype reconstruction
BLAST+ v2.12.0
CIP & CALP (https://github.com/nelumbolutea/amk_article/blob/main/6.CIP_CALP.pl)
DRIMM v1.1
MGRA v2.3.0

Custom codes available:
https://github.com/nelumbolutea/amk_article

For manuscripts utilizing custom algorithms or software that are central to the research but not yet described in published literature, software must be made available to editors and reviewers. We strongly encourage code deposition in a community repository (e.g. GitHub). See the Nature Portfolio guidelines for submitting code & software for further information.

Data

Policy information about availability of data

All manuscripts must include a data availability statement. This statement should provide the following information, where applicable:

- Accession codes, unique identifiers, or web links for publicly available datasets
- A description of any restrictions on data availability
- For clinical datasets or third party data, please ensure that the statement adheres to our policy

The datasets generated and analyzed during the current study including PacBio Sequel II, Illumina, Hi-C data, genome assembly, annotation, and RNA-seq reads have been deposited in China National GeneBank (CNGB, https://db.cngb.org/) under accession number CNP0001708. Public transcriptomes used in this study are available from NCBI under the accession number accession Nos. SRR9644796 and SRR9644797.

Field-specific reporting

Please select the one below that is the best fit for your research. If you are not sure, read the appropriate sections before making your selection.

☒ Life sciences ☐ Behavioural & social sciences ☐ Ecological, evolutionary & environmental sciences

For a reference copy of the document with all sections, see nature.com/documents/nr-reporting-summary-flat.pdf

Life sciences study design

All studies must disclose on these points even when the disclosure is negative.

Sample size	For genome sequencing and assembly of Acorus, only one individual is used to ensure the sample purity, and further low heterozygosity estimated by Kmers ensured the accuracy for genome assembly, which successfully allowed us produce a high-quality assembly. For RNA-seq, since the purpose is to confirm the expression bias towards LFs (subgenome dominance) being consistent among different tissues, a total of five tissue RNA-seq data representing different tissue types were considered as tissue replicates, and finally all tissue samples successfully concluded the same trend of LF > MF in expression.
Data exclusions	For genome sequencing, Sequel II Subreads with a quality score below 0.8 were excluded.
Replication	For genome sequencing, Sequel II generated data of 250 fold length of the Acrorus genome size, which is enough to obtain a high quality genome. For subgenome dominance tested by RNA-seq of tissues, a total of five tissues were considered as five tissue replicates, and all five samples successfully revealed the same consistent trend of LF > MF in overall expression. For phylogenetic tree of monocots and outgroups, 1000 bootstraps were used and we successfully obtained a species tree with high-confidence support.
Randomization	No randomization was applied in this manuscript since the genome assembly was not allocated into experimental groups.
Blinding	No blinding was applied in this manuscript since the genome assembly was not allocated into experimental groups.

Reporting for specific materials, systems and methods

We require information from authors about some types of materials, experimental systems and methods used in many studies. Here, indicate whether each material, system or method listed is relevant to your study. If you are not sure if a list item applies to your research, read the appropriate section before selecting a response.

Materials & experimental systems

n/a	Involved in the study
☒	☐ Antibodies
☒	☐ Eukaryotic cell lines
☒	☐ Palaeontology and archaeology
☒	☐ Animals and other organisms
☒	☐ Human research participants
☒	☐ Clinical data
☒	☐ Dual use research of concern

Methods

n/a	Involved in the study
☒	☐ ChIP-seq
☒	☐ Flow cytometry
☒	☐ MRI-based neuroimaging
